# CasMiner: a deep-learning tool for high-throughput mining and rational design of efficient Cas9

**DOI:** 10.1093/nsr/nwag090

**Published:** 2026-02-09

**Authors:** Guoshun Xu, Suzhen Li, Haoyu Li, Xiaopu Ren, Yekun Ding, Xinli Pang, Xu Liu, Qiaoling Tang, Tao Tu, Yuan Wang, Huiying Luo, Bin Yao, Jian Tian, Rumei Chen, Feifei Guan

**Affiliations:** State Key Laboratory of Animal Nutrition and Feeding, Institute of Animal Science, Chinese Academy of Agricultural Sciences, Beijing 100193, China; National Key Laboratory of Agricultural Microbiology, Biotechnology Research Institute, Chinese Academy of Agricultural Sciences, Beijing 100081, China; State Key Laboratory of Animal Nutrition and Feeding, Institute of Animal Science, Chinese Academy of Agricultural Sciences, Beijing 100193, China; National Key Laboratory of Agricultural Microbiology, Biotechnology Research Institute, Chinese Academy of Agricultural Sciences, Beijing 100081, China; National Key Laboratory of Agricultural Microbiology, Biotechnology Research Institute, Chinese Academy of Agricultural Sciences, Beijing 100081, China; National Key Laboratory of Agricultural Microbiology, Biotechnology Research Institute, Chinese Academy of Agricultural Sciences, Beijing 100081, China; State Key Laboratory of Animal Nutrition and Feeding, Institute of Animal Science, Chinese Academy of Agricultural Sciences, Beijing 100193, China; National Key Laboratory of Agricultural Microbiology, Biotechnology Research Institute, Chinese Academy of Agricultural Sciences, Beijing 100081, China; State Key Laboratory of Animal Nutrition and Feeding, Institute of Animal Science, Chinese Academy of Agricultural Sciences, Beijing 100193, China; State Key Laboratory of Animal Nutrition and Feeding, Institute of Animal Science, Chinese Academy of Agricultural Sciences, Beijing 100193, China; State Key Laboratory of Animal Nutrition and Feeding, Institute of Animal Science, Chinese Academy of Agricultural Sciences, Beijing 100193, China; State Key Laboratory of Animal Nutrition and Feeding, Institute of Animal Science, Chinese Academy of Agricultural Sciences, Beijing 100193, China; State Key Laboratory of Animal Nutrition and Feeding, Institute of Animal Science, Chinese Academy of Agricultural Sciences, Beijing 100193, China; National Key Laboratory of Agricultural Microbiology, Biotechnology Research Institute, Chinese Academy of Agricultural Sciences, Beijing 100081, China; State Key Laboratory of Animal Nutrition and Feeding, Institute of Animal Science, Chinese Academy of Agricultural Sciences, Beijing 100193, China; National Key Laboratory of Agricultural Microbiology, Biotechnology Research Institute, Chinese Academy of Agricultural Sciences, Beijing 100081, China

**Keywords:** CRISPR-Cas9, deep learning, CasMiner, VpCas9, editing efficiency

## Abstract

Since its inception, the CRISPR-Cas system, particularly Cas9, has demonstrated immense potential for life science applications, but expansion of the Cas9 toolkit is constrained by sequence-alignment-based strategies for mining and optimization. Here, we developed CasMiner—a deep-learning model for discovering and engineering novel Cas9 proteins. CasMiner achieved 99.63% accuracy in predicting Cas9s and identified VpCas9 from public databases. Experimental validation showed that VpCas9 exhibits robust double-strand cleavage activity. Combining CasMiner and evolutionary analysis, we engineered three mutants with markedly increased structural rigidity and positive charge. *In vivo* cleavage assays revealed that the mutant VPM2-3 achieved a higher average editing efficiency in rice callus and maize protoplasts than the wild-type VpCas9, the editing efficiency of which rivals that of SpCas9. This study thus establishes a comprehensive platform for mining and engineering Cas9 proteins, and provides VpCas9 and derivative nucleases as powerful tools that greatly broaden the horizon for genome-editing applications.

## INTRODUCTION

Originally identified as an adaptive immune mechanism in prokaryotes [1,2], the CRISPR-Cas system has been broadly developed for genome editing in animals [3,4] and plants [5,6], with Cas9 serving as the most well-studied and widely used core nuclease component [1,6]. Despite remarkable progress in genome-editing technologies, the vast majority of tools, e.g. Base Editor [7,8] and Prime Editor [7,9], continue to heavily rely on Cas9 for recognition and cleavage activity [8,9]. Therefore, research on the CRISPR-Cas9 system continues to advance.

In recent years, most research on Cas9 has focused on its application in different systems and targets, such as molecular crop breeding, in which gene knockout [10,11], knock-in [12,13] or altered regulation [14,15] through genome editing have led to improved yield and crop quality. However, the current, extensively utilized Cas9 proteins, predominantly SpCas9, still require further improvement of their off-target activity and editing efficiency for many high-precision applications [16], such as in therapeutic development, although relatively few studies have focused on the mining and design of advanced Cas9 tools. Furthermore, at present, data mining for Cas9 candidates, e.g. Nme2Cas9, Nme3Cas9 [17] and ScCas9 [18], continues to primarily rely on homology-based or similarity-based searches. This process involves multiple rounds of BLAST 2.15.0+searches to accommodate the high diversity of Cas9 sequences [19]. However, the diversity of query sequences, subjective parameters for sequence quality and the biased or arbitrary selection of representative clusters all influence the outputs of sequence mining, thus imposing clear limitations that may lead to the loss of potentially valuable Cas9 proteins [1]. Furthermore, in a broad sense, Cas9 proteins perform remarkably similar DNA recognition and cleavage functions despite widely varying amino acid sequences [20,21]. It is thus reasonable to speculate that important characteristics other than the known conserved domains may be in the Cas9 protein sequence, which might be easily overlooked with the straightforward homology or similarity comparisons utilized by current methods.

More recently, deep-learning models have shown great potential for learning and extracting protein-sequence characteristics, and such models have been employed to annotate functional motifs in protein sequences, such as transmembrane regions, sorting signals or lipidation and phosphorylation sites [22]. With respect to CRISPR systems, a number of deep-learning models have been developed to design guide Ribonucleic acids (RNAs) [23,24] or predict editing efficiency [25,26] and off-target rates [27,28]. For example, Liu and co-workers collected 4790 results of target sequence repair to construct the Apindel model for predicting CRISPR-Cas9-mediated genetic variation repair outcomes, which had area-under-curve (AUC) values of >90% in predicting deletion frequency and 1-bp insertion frequency in target sequences [26]. Additionally, Ruffolo *et al.* [29] have developed an artificial-intelligence-generated gene editor, OpenCRISPR-1, which demonstrates activity and specificity comparable to those of SpCas9. However, models for mining Cas9 proteins are seldom reported.

Here, we developed a deep-learning model, CasMiner, to facilitate the mining and design of new Cas9 proteins. CasMiner could learn and visualize specific characteristics of the Cas9 sequence, which enabled our discovery and subsequent modification of an unreported functional Cas9 protein, VpCas9. Using a fluorescent reporter in *Escherichia coli* with *in vitro* experiments, we found that VpCas9 had comparable editing efficiency to SpCas9. Through mutation design with CasMiner, we obtained three double mutants that displayed higher editing efficiency than wild-type *in vitro*. VpCas9 and its engineered variants also demonstrated high efficiency and specificity for genome editing in diverse systems, including rice callus, stably transformed rice plants, maize (*Zea mays*) protoplasts and mammalian HEK293T cells. In rice callus and maize protoplasts, the activity of VpCas9 is comparable to that of SpCas9, with all three mutant variants outperforming the wild-type. This study thus provides a powerful platform for mining Cas9 proteins from large-scale protein databases, as well as an effective candidate gene editor that may be further developed for biomedical research, therapeutic or agricultural applications.

## RESULTS

### Construction, evaluation and characteristics extraction of CasMiner

**Figure 1.**
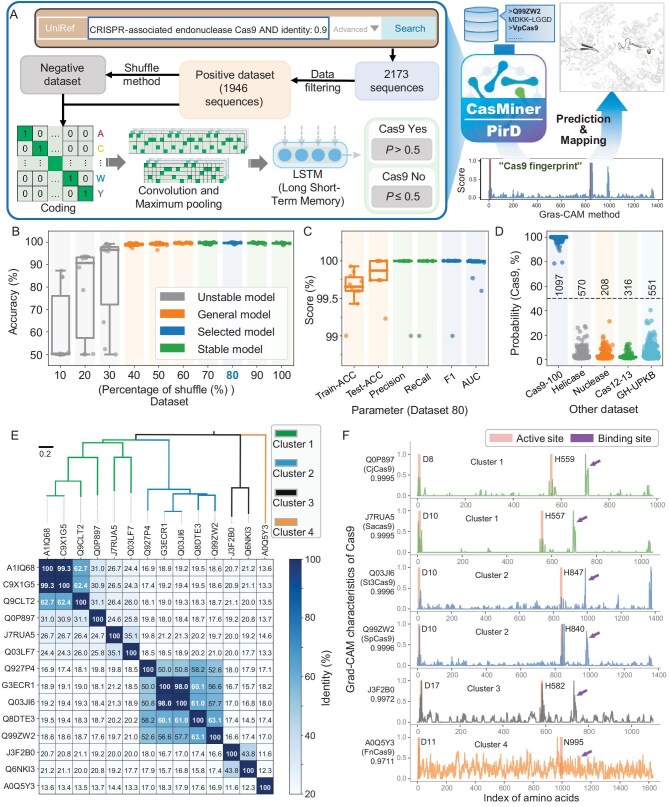
CasMiner construction, performance evaluation, Cas9 sequence analysis and Grad-CAM analysis. (A) Workflow for data collection, model training and model evaluation to construct CasMiner. (B) Model accuracy following training with different percentage thresholds for sequence shuffling. (C) Overall performance of the Dataset80 model (CasMiner) evaluated by Training set accuracy (Train-ACC), Test set accuracy (Test-ACC), Precision, Recall, F1 and AUC. (D) CasMiner performance on the helicase dataset, the nuclease dataset, the Cas12-Cas13 dataset, the GH-UPKB dataset and the UniRef100 Cas9 dataset, excluding UniRef90. (E) Pairwise comparison of sequence identity among 14 experimentally validated Cas9s based on sequence alignment and phylogenetic clustering. (F) Grad-CAM extraction of sequence characteristics in the 14 validated Cas9 sequences. In (F), the vertical bars (active centers) and arrows (binding sites) indicate significant sequence characteristics extracted by CasMiner.

In order to improve efficiency in the mining, design and optimization of core endonuclease in the CRISPR-Cas9 toolbox, specifically the Cas9 proteins, we first trained a deep-learning model (Fig. 1A) with a dataset of 1946 protein sequences annotated as Cas9 nucleases in the UniRef90 database, ranging from 801 to 1820 AA in length (i.e. the positive dataset). To provide a learning context for the model, we then generated datasets containing shuffled (i.e. randomized) sequences from the positive dataset (i.e. negative datasets), in which the amino acid frequencies remained intact, but potential characteristics hidden in the sequence were thoroughly disrupted [30]. Specifically, shuffling thresholds of 10%∼100%, in 10% intervals, were used to rearrange the positive amino acid sequences, resulting in 10 total negative datasets (Dataset10 through Dataset100). Each negative dataset and the positive dataset were then used as inputs to train a convolutional neural network and long short-term memory (CNN-LSTM) network-based framework, thus yielding 10 different models (Fig. 1A and Fig. S1).

To subsequently evaluate the accuracy of these 10 models in predicting nuclease function, we calculated several evaluation parameters. This analysis revealed that the prediction accuracy increased along with the percent shuffling threshold in the negative datasets (Fig. S2) with the model trained on Dataset80 (the negative dataset) and the positive dataset demonstrating the highest training accuracy (99.63 ± 0.25%, Fig. 1B). Additionally, the Dataset80-based model also showed strong performance on the test set, with accuracy, precision, recall, F1 value and AUC scores all close to 100% (Fig. 1C and Fig. S2).

To further evaluate the generalizability of the 10 models, we also constructed a UniRef100 Cas9 dataset (Cas9-100; 1097 sequences), a nuclease dataset (208 sequences), a Cas12 and Cas13 dataset (Cas12-13; 316 sequences), a helicase dataset (570 sequences) and a glycoside hydrolase (GH) dataset (GH-UPKB; 551 sequences). Classification with each of the 10 models revealed clear differences in the predictive performance among models on each of these datasets (Fig. S3). Notably, the Dataset80 model again achieved the highest prediction accuracy (99.77 ± 1.13%) for Cas9s in the alternative dataset (Fig. S3A), with a low probability of predicting Cas9 sequences in non-Cas9 datasets (3.40 ± 2.92% in the helicase dataset; 3.41 ± 3.00% in the nuclease dataset; 2.46 ± 1.52% in the Cas12-13 dataset; and 4.81 ± 4.78% in the GH dataset; Fig. 1D and Fig. S3B–E), thus confirming its strong generalizability. Given these results, we designated this model as ‘CasMiner’ and applied it in subsequent analyses.

Given its strong performance in the above prediction tasks, we next explored whether CasMiner learned characteristics within the Cas9 protein sequences. To this end, we first compiled the 14 experimentally validated Cas9 sequences in the UniProt-Swiss-Prot database (2024.10.10). A similarity matrix based on sequence alignment and phylogenetic analysis showed that 14 Cas9 sequences clustered into four categories (Fig. 1E). Pairwise comparisons showed generally low sequence identity between Cas9s, with 68 (74.7%) pairs sharing <25% identity and only two (2.2%) pairs having >90% identity (Fig. 1E and Fig. S4). Notably, CasMiner could successfully predict all of these sequences as Cas9s (Fig. S5).

Following these predictions, we applied each Cas9 and non-Cas9 (Cas12 and Cas13, nuclease, helicase and GH) sequence as inputs for CasMiner and gradient-weighted class activation mapping (Grad-CAM) was used to extract and downscale the convolutional layer matrix [31]. Examination of the resulting signal intensity peaks uncovered a set of regular peaks exclusively present in the Cas9 sequences, which we designated as the Cas9 fingerprint, whereas no such set of regular peaks could be observed in the non-Cas9 sequences (Fig. 1F and Figs S5 and S6). These results suggested that CasMiner could extract and learn the distinct characteristics of Cas9 protein sequences. In particular, this analysis indicated that all Cas9 fingerprints contained two prominent peaks associated with their respective active sites. For example, the characteristic peaks of SpCas9 (Q99ZW2) were close to its active sites (D10 and H840) (Fig. 1F and Fig. S5), while FnCas9 (A0Q5Y3) had a characteristic peak at a conserved potential active site, H969 [20,32] (Fig. 1F and Fig. S7). In addition to these peaks associated with active sites, most Cas9 fingerprints had peaks associated with binding sites, such as H983 in SpCas9 [20] and H701 in SaCas9 [32] (Fig. 1F and Fig. S5).

### Identification of VpCas9 through genome mining with CasMiner

**Figure 2.**
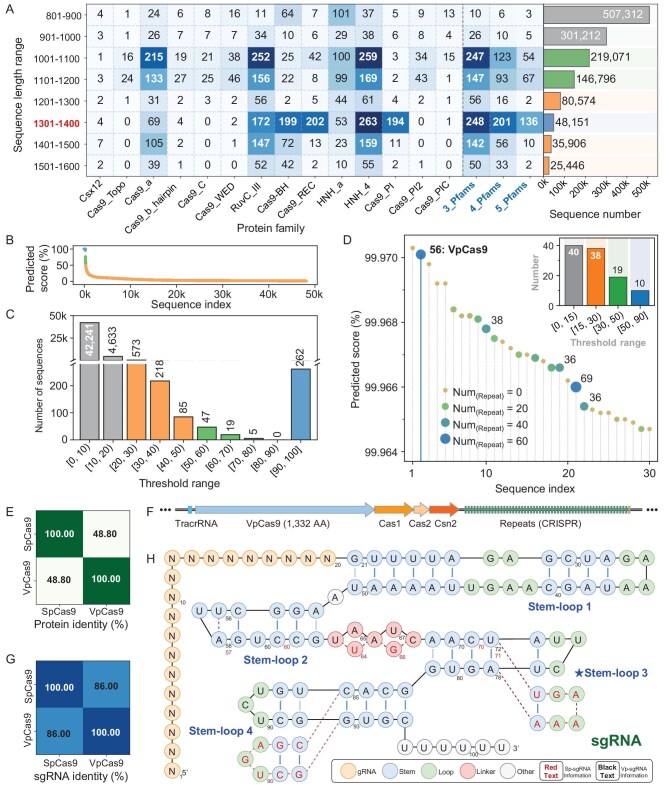
Discovery of VpCas9 and sgRNA construction. (A) Representative protein datasets with Pfam annotations based on length thresholds (left) and statistics of sequence entries in each dataset (right). (B) Overall prediction results of CasMiner. (C) Statistical summary of sequence entries at different CasMiner prediction score thresholds. (D) Distribution of repeats among the top 30 sequences based on CasMiner score and statistical summary of sequence entries under different repeat number thresholds. (E) Protein-sequence identity between VpCas9 and SpCas9. (F) Diagram of CRISPR-Cas9-related gene elements in the *V. penaei* CD276T genome. (G) SgRNA sequence identity between VpCas9 and SpCas9. (H) Comparison of sequence and secondary structures between Vp-sgRNA and Sp-sgRNA. Detailed information for sequence alignments in (E) is presented in Supplementary material 1; and (F) specific sequence information is provided in Supplementary material 2.

Despite the effectiveness of CasMiner in extracting the distinguishing characteristics from the Cas9 sequence, directly mining Cas9 candidates from non-redundant genome collections (https://progenomes.embl.de/download.cgi) presents a challenge due to the coarse annotation of the Cas9 proteins in the whole dataset. To narrow down the dataset, we assembled a dataset containing only genomic information encoding proteins from 800 to 1600 residues in length. The remaining 1.36 million proteins were further divided into eight distinct subsets based on 100-AA size increments and annotated by searching against the Pfam database (Table S1) using HMMER [33]. This annotation process showed that protein sequences ranging from 1301 to 1400 AA had the highest number of entries containing simultaneous annotations for multiple Cas9-related Pfam domains. Specifically, 248 sequences in this cluster contained three Pfams, 201 had four Pfams and 136 contained five Pfams. This subset contained the highest frequency of RuvC_III (172), Cas9_BH (199), Cas9_REC (202), HNH_4 (263) and Cas9_PI (194) annotations (Fig. 2A). Collectively, these results suggested that this dataset had a higher likelihood of containing active Cas9 proteins and it was therefore selected for subsequent screening with CasMiner.

Scanning this dataset with CasMiner identified 333 sequences with a prediction score of >50%, 262 of which achieved a prediction score of ≥90% (Fig. 2B and C, and Table S2). After successfully downloading 214 genomes, corresponding to 218 potential Cas9 sequences, we searched for repeat sequences, as previous studies have shown that a higher repeat number correlates well with the presence of the CRISPR-Cas system in different species [34,35]. Analysis by using the CRISPR Recognition Tool identified 107 Cas9 proteins associated with repeat sequences in close genomic proximity (upstream or downstream) [36], 10 of which were associated with >50 repeat sequences. Finally, as no repeat sequences were detected in the genome harboring the top-scoring candidate, we selected the sequence with the second-highest CasMiner score, which contained 56 repeats (2/107, Fig. 2D and Table S2). The sequence was derived from the *Vagococcus penaei* strain CD276T genome and was subsequently designated as VpCas9 for subsequent characterization.

Sequence analysis indicated that VpCas9 comprised 1332 amino acids and had 48.8% sequence identity with SpCas9 (Fig. 2E) [20]. Further examination of the *V. penaei* CD276T genome revealed that coding sequences of Cas1, Cas2 and Cns2 were present in the downstream region of VpCas9 as repeat-containing CRISPR arrays, all of which are classical characteristics of the CRISPR-Cas9 system. In addition, BlastN analysis of the repeat sequences adjacent to VpCas9 showed high identity with TracrRNA. We subsequently designed and synthesized a corresponding sgRNA (Fig. 2F), which shared 86.0% identity with the SpCas9 sgRNA (Fig. 2G). This finding suggested that, despite their relatively large differences in amino acid composition, VpCas9 targeted a highly similar sgRNA to that of SpCas9 (Fig. 2E and G, and Fig. S8). Further analysis of the differences in the repeat, TracrRNA and sgRNAs between VpCas9 and SpCas9 indicated that the most prominent differences were primarily concentrated at the 3' end of the TracrRNA and sgRNA (Fig. S8). This evidence, together with secondary structure analysis, revealed that differences in the Sp-sgRNA and Vp-sgRNA sequences were mainly located in the Linker, Stem-loop3 and Stem-loop4 regions, and resulted in a compact Stem-loop3 used by SpCas9 (Fig. 2H).

### Characterization of VpCas9 PAM (Protospacer Adjacent Motif) preference and activity

**Figure 3.**
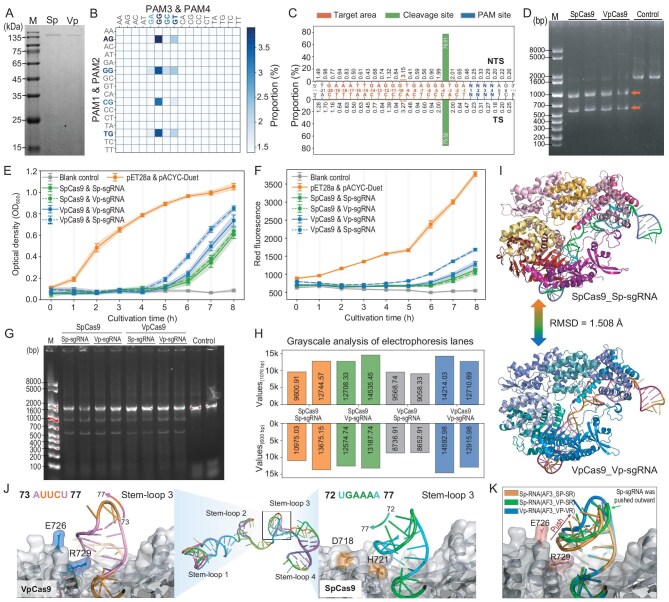
Cleavage mode of VpCas9 and structural comparison with SpCas9. (A) Protein expression and purification of SpCas9 and VpCas9. (B) Analysis of VpCas9 PAM preference using a PAM library. (C) Analysis of VpCas9 preferential cleavage sites. (D) *In vitro* cleavage of short fragments by VpCas9 and SpCas9. (E, F) *In vivo* growth–fluorescence detection assays of gene-editing efficiency by the VpCas9 and SpCas9 systems. (G) Electrophoretic separation of cleavage products from the same nucleic acid substrate to assess the reciprocal compatibility of sgRNAs between VpCas9 and SpCas9 *in vitro*. (H) Quantified by grayscale analysis. (I) AlphaFold3 predictions and comparison of the CRISPR-SpCas9&Sp-sgRNA and CRISPR-VpCas9&Vp-sgRNA holostructures. (J) Structural comparison between Vp-sgRNA and Sp-sgRNA, and differences in stem–loop 3 conformation. (K) Protein-structure alignments comparing sgRNA docking between Cas9s.

To confirm that VpCas9 exhibited Cas9 nuclease function, we heterologously expressed it in *E. coli* (Fig. 3A). Examination of possible PAM preference by using a PAM library containing 256 NNNNs (Fig. S9A) showed that VpCas9 had an NGGV PAM (N: A/T/C/G, V: G/T) and the first three bases of the NGG in this PAM were the same as those of SpCas9 [37]. At the same time, we found that VpCas9 exhibits an obvious preference for guanine (G), followed by thymine (T), at the fourth position of the PAM, thus introducing an additional base constraint (Fig. 3B and Fig. S9B). Analysis of the sequencing fragments showed that, similarly to SpCas9, VpCas9 exhibited significant double-strand cleavage activity at the –3 to –4 nt position upstream of the PAM, mediating cleavage in 75.52% of the target sequence (TS) and 76.91% of the non-target sequences (Fig. 3C and Fig. S9C). We also designed and synthesized an sgRNA targeting a nucleic acid fragment that could be cleaved into 600- and 1057-bp fragments to quantify VpCas9 and SpCas9 cleavage activity *in vitro* (Fig. S9D and Table S3). Electrophoretic separation of the resulting fragments showed that both VpCas9 and SpCas9 could cleave the substrate sequence (Fig. 3D), further supporting the VpCas9 function as an RNA-guided endonuclease for precise dsDNA cleavage.

To further validate the genome-editing activity of VpCas9 in *E. coli*, we constructed a fluorescence reporting system (BMLacZ; *mApple* inserted into the *LacZ* gene of *E. coli* BL21(DE3), Fig. S9E–F). As the lack of a repair system in *E. coli* leads to cell death and reduced fluorescence intensity upon genomic editing, we used the growth density and fluorescence signal of the engineered bacteria to assess the genomic editing activity. For this purpose, a plasmid harboring VpCas9 or SpCas9 was co-transformed into *E. coli* together with a plasmid containing the corresponding sgRNAs. Transformation with either CRISPR-VpCas9&Vp-sgRNA or CRISPR-SpCas9&Sp-sgRNA resulted in a reduced growth rate and fluorescence intensity (Fig. 3E and F), suggesting genomic editing activity. To assess the similarities between the two Cas9 sgRNA sequences (Fig. 3E and F), the sgRNAs for the two systems were swapped. Comparison between the recombinant *E. coli* strains expressing the CRISPR-VpCas9&Sp-sgRNA and CRISPR-VpCas9&Vp-sgRNA systems showed that the latter system induced slower growth and lower fluorescence intensity in the host bacteria, indicating higher editing activity. In contrast, the CRISPR-SpCas9 system had a negligible difference in activity between the Sp- or Vp-sgRNAs (Fig. 3E and F), suggesting no preference for either sgRNA. Moreover, VpCas9 and SpCas9 could use each other’s sgRNA to cleave the same nucleic acid fragment *in vitro* (Fig. 3G), while band-density analysis of the cleavage products indicated that CRISPR-VpCas9&Vp-sgRNA exhibited higher activity than the CRISPR-VpCas9&Sp-sgRNA system (Fig. 3H), suggesting that Vp-sgRNA could mediate higher cleavage activity than Sp-gRNA.

Comparison of the predicted VpCas9 and SpCas9 protein structures by using AlphaFold3 [38] yielded an root mean square deviation (RMSD) score of 1.508 Å between the structures, while the two predicted sgRNA structures similarly exhibited high consistency (Fig. 3I), which could at least partially explain the reciprocal compatibility of the sgRNAs between these systems. Further examination of the SpCas9 and VpCas9 holostructures bound with their respective sgRNAs confirmed that the stem-loop3 region of Vp-sgRNA indeed formed a taller and leaner structure than that of Sp-sgRNA. Specifically, the A73-U77 bases in loop 3 of Vp-sgRNA were positioned farther from the protein surface compared with the U72-A77 counterpart region in loop 3 of Sp-sgRNA, which instead extended toward the surface of SpCas9 (Fig. 3J). Correspondingly, the E726 and R729 residues in VpCas9 formed a cleft that fitted the extended stem–loop 3 region, but pushed away the planoconcave Sp-sgRNA stem–loop 3 structure. In contrast, SpCas9 residues D718 and H721 participated in forming a planar surface that could readily accommodate either Vp- or Sp-sgRNAs (Fig. 3J and K, and Fig. S10). Therefore, we revealed the specific interaction mechanism between the extended conformation of Vp-sgRNA stem–loop 3 and the surface cracks of VpCas9 by structural analysis and explained its activity advantage.

### CasMiner-assisted design of VpCas9 variants

**Figure 4.**
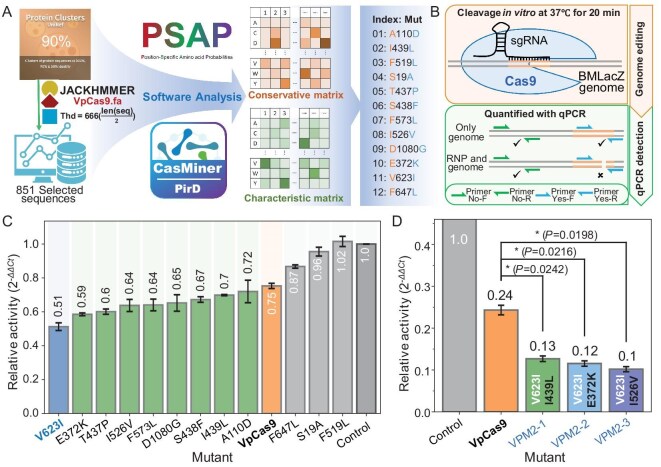
VpCas9 mutant design and qPCR-based evaluation of editing activity. (A) Twelve single residue conversions were designed by using a characteristic matrix of VpCas9 generated with CasMiner in conjunction with a conservation matrix derived from PSAP. (B) A qPCR-based approach for detecting genomic target-site cleavage to assess VpCas9 editing efficiency. (C) Editing activity of 12 single-mutant VpCas9 variants. (D) Editing activities of VpCas9 double mutants. Data show means ± STD and significance was determined by using a two-tailed *t*-test at the level of *; *P* < 0.05, significant; ns, not significant.

To further improve the efficiency of the VpCas9 cleavage activity, CasMiner was applied to design a mutation strategy for VpCas9. For this purpose, 851 sequences for VpCas9 homologs were acquired from the UniRef90 database and characteristic matrices of the homologous sequences were extracted from the convolutional layer of CasMiner. A core site matrix was then obtained by processing the homologous characteristic matrix (Table S4). At the same time, a conservative matrix was obtained by calculating position-specific amino acid probabilities (PSAP), which were then used to analyse evolutionary trends in the above core sites (Table S5). We subsequently calculated the difference (Diff) scores between the mutant amino acids and original amino acids in the CasMiner-derived characteristic matrix and the PSAP-derived conservation matrix, and ranked these scores from high to low (see ‘Materials and methods’). Finally, we compared the ranks of the mutant and conserved sites and ultimately identified 12 target sites for mutation that each had a total ranking score of <30 (Fig. 4A and Table S6).

In order to improve the accuracy in determining the *in vitro* editing efficiency of the VpCas9 mutants, we developed a quantitative real-time PCR (qPCR)-based method in which the sgRNAs targeted a genomic copy of BMLacZ in a recombinant *E. coli* strain. Primers were then designed to amplify a 203-bp region containing the cleavage site, while an additional primer pair was designed to cut an adjacent, non-targeted region that served as the reference locus. The relative editing efficiency of the VpCas9 variants was then calculated by using the ${2}^{ - \Delta \Delta Ct}$ method, with a higher ${2}^{ - \Delta \Delta Ct}$ at the BMLacZ edit site indicating less cleavage activity (Fig. 4B). Among the 12 VpCas9 variants, 9 showed higher editing efficiency than the wild-type, with the VpCas9^V623I^ variant demonstrating the highest activity (Fig. 4C). Based on these results, we selected the VpCas9^V623I^ background for further mutation at other sites that yielded higher activity than the wild-type in the single mutants. Among the eight resulting double mutants, we found that VpCas9^V623I-I439^^L^ (VPM2-1), VpCas9^V623I-E372K^ (VPM2-2) and VpCas9^V623I-I526V^ (VPM2-3) showed significantly higher editing efficiency than VpCas9 (Fig. 4D and Fig. S11).

### Molecular dynamics analysis of the mechanism underlying increased activity

**Figure 5.**
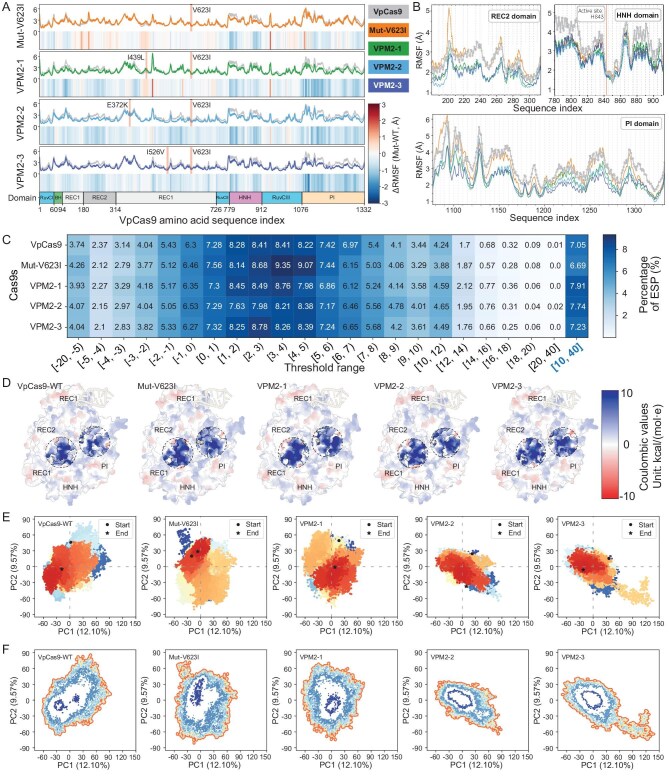
MD analysis of VpCas9 and its mutants. (A, B) Overview of RMSF in VpCas9 and each high efficiency variant. (C) The percentage of different coulombic electrostatic potential (ESP) thresholds was calculated following protein stabilization in MD simulations; and (D) ESP was mapped to tertiary protein structures. (E) PCA and (F) free-energy landscape analysis of VpCas9 and its mutants.

To investigate the possible mechanisms responsible for the increased editing activity in the variants, we performed molecular dynamics (MD) analysis of the 500-ns trajectories for VpCas9, VpCas9^V623I^ and the three double mutants. The results showed that the RMSD stabilized after 400 ns (Fig. S12A), reaching a dynamic equilibrium state. Further analysis of the root mean square fluctuation (RMSF) values showed that all mutants exhibited reduced fluctuation in the His-Asn-His endonuclease (HNH) domain compared with the wild-type (Fig. 5A). Additionally, fluctuations in the PAM-interacting (PI) domain and REC2 domain of VPM2-1, VPM2-2 and VPM2-3 were also reduced compared with the wild-type VpCas9 and VpCas9^V623I^ (Fig. 5B). The decrease in the fluctuation of these three structural domains suggested that the structural rigidity had increased, potentially facilitating the recognition and cleavage of target sites, and thereby enhancing the editing efficiency [39].

RMSF analysis of amino acid residues near the mutation site revealed that the four sites involved in the mutation were all in the REC1_2 domain, relatively far from the HNH and PI domains (Fig. S12B), and no significant change in the structural rigidity was observed near the mutation sites (Fig. S12C–E). These results suggested that mutation of these sites mainly regulated the protein structure and dynamics through long-range effects that improved their activity and stability [40]. Exploring this possibility, analysis of the electrostatic potential by using structures extracted from the last frame of the MD showed that more positive charges were present on the surface of all proteins. VPM2-1, VPM2-2 and VPM2-3 had 12.25%, 9.79% and 2.54% higher positive charge ratios than VpCas9, respectively (Fig. 5C), indicating that these mutant Cas9 proteins can attract negatively charged nucleic acids faster. Furthermore, VpCas9^V623I^, VPM2-1, VPM2-2 and VPM2-3 had stronger positive charges near the channel entrance of the HNH and REC1 domains or in the inner wall of the cavity surrounded by the PI and REC1 domains compared with the wild-type (Fig. 5D and Fig. S13). These results indicated that these changes enhanced the interaction between the Cas9 mutant and negatively charged DNA TS, thus improving the editing efficiency.

In addition, dimensionality reduction principal component analysis (PCA) of the protein alpha carbons for each repetition of each frame revealed that PC1 could explain 12.10% of the variation while PC2 was responsible for 9.57%. It was observed that the scatter distribution of the mutants was denser, indicating that the conformational changes in the mutants were smaller than those of the wild-type, which is consistent with the results of the RMSD (Fig. 5E). Free-energy landscape analysis based on PCA indicated that the areas of minimum free energy were substantially larger in the VpCas9^V623I^ (377.40 Å^2^), VPM2-1 (570.17 Å^2^), VPM2-2 (1050.41 Å^2^) and VPM2-3 (987.88 Å^2^) mutants compared with those in wild-type VpCas9 (315.37 Å^2^, Table S7), indicating that these mutations resulted in merging separate low-free-energy regions into a single, larger region most notably in VPM2-2 and VPM2-3 (Fig. 5E). The expansion of such energy depressions in the variants indicates that they have higher structural stability in the solvent and are more likely to enter the low-energy state.

### Application of VpCas9 and mutants in different organisms

**Figure 6.**
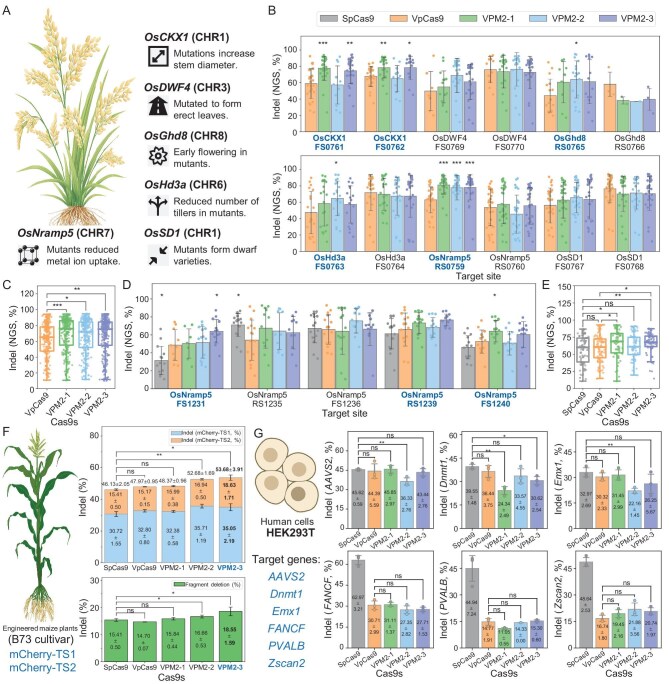
Application of VpCas9 and mutants for genome editing in rice callus, maize protoplasts and HEK293T cells. (A) Genes selected for editing in rice callus. (B) Gene-editing efficiency at two target sites in each gene. (C) Average gene-editing efficiency across all targets and loci. (D) Comparison of editing efficiency with SpCas9 on five targets of *OsNramp5*. (E) Average gene-editing efficiency across these five targets. (F) Gene-editing efficiency at two target sites in maize protoplasts (cultivar B73). (G) Gene-editing efficiency at six targets across six genes in HEK293T cells. For (B) and (C), 32 replicates were used; for (D) and (E), 16 replicates were used; and for (F) and (G), 3 replicates were used. In (B) and (D), the bold-labeled loci are mutants with a increase in editing efficiency over the wild-type. In addition, significance was determined by using a two-tailed *t*-test at the level of ***, *P* < 0.001, extremely significant; **, *P* < 0.01, highly significant; *, *P* < 0.05, significant; ns, not significant.

To evaluate the application potential of VpCas9 and its mutants, we selected rice, maize and mammalian HEK293T cells to assess the editing efficiency and off-target rates. In rice, 12 target sites across six genes with known differences in quantitative traits, such as stem diameter (*OsCKX1*) [41], leaf erectness (*OsDWF4*) [42], flowering time (*OsGhd8*) [43], tiller number (*OsHd3a*) [44], ion absorption (*OsNramp5*) [45] and plant height (*OsSD1*) [46], were selected (Fig. 6A). Using the *Agrobacterium*-mediated transformation of rice calluses, next-generation sequencing (NGS) analysis to quantify indels revealed editing efficiencies ranging from 37.00% to 81.20%. Notably, the mutants had significantly higher editing efficiency than the wild-type at five targets, including OsCKX1-FS0761, OsCKX1-FS0762, OsGhd8-RS0765, OsHd3a-FS0763 and OsNRAMP5-RS0759, with VPM2-1 (81.20 ± 11.05%), VPM2-2 (77.79 ± 14.94%) and VPM2-3 (78.23 ± 15.36%) showing the greatest increase at OsNRAMP5-RS0759 compared with VpCas9 (63.08 ± 16.05%, Fig. 6B and Table S8). Averaging the editing efficiencies across the sites further emphasized this increase in editing performance of the VPM2-1 (68.46 ± 21.78%), VPM2-2 (65.88 ± 22.51%) and VPM2-3 (67.12 ± 22.57%) mutants relative to VpCas9 (61.35 ± 22.02%; Fig. 6C and Table S8). Additionally, off-target effects were nearly undetectable at the examined potential off-target sites (Table S9). Further analyses revealed that all three mutants exhibited comparable or higher editing rates in callus than the wild-type at most target sites (Supplementary result 1, Fig. S14A and Table S10). Additionally, analysis of the editing types (indels) at each site indicated that VpCas9 and its mutants could introduce either insertions or deletions (Fig. S14B). To further characterize the editing performance, VpCas9 and its mutants were assessed across the same 12 target sites in stably transformed rice plants regenerated from the initial callus. Figs S14C–E shows that the overall editing efficiency was comparable between regenerated plants and callus tissue, although the OsGhd8 locus remained the most refractory to editing. Average efficiencies ranged from 61.95 ± 26.99% to 70.12 ± 22.14%, with VPM2-3 demonstrating the highest activity (Table S11, S12), aligning with previously reported trends [47].

Next, to benchmark VpCas9 and its mutants against SpCas9, we designed an additional five target sites within the editable gene *OsNramp5* and evaluated their editing efficiency in rice callus tissue. NGS results revealed that across all five target sites, the average editing efficiency of VpCas9 was comparable to that of SpCas9. Notably, the editing efficiencies of VPM2-1 (65.23 ± 19.76%) and VPM2-3 (66.03 ± 15.48%) were significantly higher than those of both VpCas9 (58.23 ± 19.24%) and SpCas9 (56.06 ± 20.96%; Fig. 6D and E, and Table S13). Additionally, off-target analysis targeting five potential off-target sites per target locus revealed no detectable editing at the majority of the sites examined. However, at three specific sites, including FS1231-OTS 5, FS1236-OTS 3 and RS1239-OTS 1, measurable off-target editing was observed for all Csa9s tested. The three VpCas9 mutants exhibited off-target rates that were comparable to that of SpCas9 (Fig. S15 and Table S14).

In maize, two target sites were designed on the *mCherry* reporter gene integrated into the genome to detect the effeteness of the VpCas9 and its mutants. The results showed that VpCas9 (total editing efficiency: 47.97 ± 0.95%; fragment knockout efficiency: 14.70 ± 0.07%) and SpCas9 (total editing efficiency: 46.13 ± 2.05%; fragment knockout efficiency: 15.41 ± 0.50%) exhibited comparable editing efficiencies, as well as similar fragment knockout efficiencies between the two targets. In contrast, the mutant VPM2-3 (total editing efficiency: 53.68 ± 3.90%; fragment knockout efficiency: 18.55 ± 1.60%) demonstrated significantly higher editing efficiencies in both respects compared with VpCas9 and SpCas9 (Fig. 6F, Fig. S16A and Table S15). Off-target analysis, examining six potential off-target sites per target locus, showed trends that were similar to those observed in rice callus: the majority of the sites exhibited low or undetectable off-target activity. At susceptible loci, VpCas9 and its variants performed comparably to SpCas9 (Fig. S16B and Table S16).

In mammalian HEK293T cells, we assessed the on- and off-target editing efficiencies of VpCas9 and its mutants across six genomic loci. The results demonstrated that VpCas9 and its mutants could edit all targets tested. Although the editing efficiencies were lower than those of SpCas9 at the targets of *FANCF, PVALB* and *Zscan2*, they reached comparable levels to those of SpCas9 at the targets of *AAVS2, Dnmt1* and *Emx1* (Fig. 6G and Table S17). Notably, in off-target assays at the AAVS2_OTS_219 and FANCF_OTS_256 target sites, SpCas9 exhibited off-target rates of 51.31 ± 0.60% and 23.57 ± 2.45%, respectively, while VpCas9 or its mutants exhibited no detectable off-target editing. At the other tested sites, the off-target rates were negligible or undetectable (Fig. S17 and Table S18).

## DISCUSSION

In this study, to systematically evaluate the performance of CasMiner, we conducted comprehensive comparisons and discussions across three key dimensions: (i) generalization capability evaluations against other models trained on shuffled-sequence negative dataset (MP-TRANS [48], ESM2 [49], RF and SVM; Figs S18 and S19, and Table S19, and see Supplementary Result 2); (ii) performance assessments of the models trained on an authentic-sequence negative dataset (Fig. S20 and Table S20, and see Supplementary Result 3); (iii) evaluation of the predictive performance of CasMiner on an extended real-world dataset containing 32 487 non-Cas9 sequences (Fig. S21 and Table S21, and see Supplementary Result 4). Through these comparative analyses, we demonstrated that CasMiner accurately identifies Cas9 proteins from both shuffled sequences and authentic non-Cas9 sequences, outperforming all the other models. These generalization-ability results suggested the CasMiner could effectively recognize these sequence-based characteristics (e.g. correct functional site and complete functional domains) as required for functioning in natural Cas9 sequences outside the training set. This capability could be used to circumvent data-sparsity and data-imbalance problems in model-based research. However, to our knowledge, the proportion of internal sequence shuffling is a critical parameter influencing the effectiveness of the method. In the study by Yu *et al.* [30], a completely random rearrangement was used, whereas, in our work, this proportion was validated to be 80% (Fig. S3). Therefore, when constructing new models by using the internal sequence shuffling approach, it is essential to systematically evaluate and select the optimal shuffling percentage from scratch.

As CasMiner relies on sequence characteristics to identify Cas9 proteins, we compared its ability to those of conventional sequence retrieval tools (BlastP and HMMER). Notably, BlastP, showed high coverage when screening with a query from the same cluster Cas9 (i.e. Cluster1 Cas9 > 66%, Cluster2 > 98%), but showed markedly lower coverage when querying with other cluster Cas9 (i.e. reciprocal searches with Cluster2 or Cluster1: <55% coverage) or missed other types altogether (e.g. Cluster4 Cas9 sequences were isolated and other types were absent; Fig. S22A). Alternatively, using a set of 14 Pfam domains, HMMER could annotate different Cas9 based on three to seven different Pfams, only one of which, the HNH_4 domain (3%–6% of the full-length sequence), was universal to all Cas9 proteins (Fig. S22B). Overall, it is challenging for BlastP and HMMER to retrieve more Cas9 sequences when using a specific Cas9 sequence or a Pfam query. Furthermore, in the evaluation of computational resource consumption and runtime across different tools (Fig. S23 and Table S22, and see Supplementary Result 5), we found that, compared with other models, CasMiner, BlastP and HMMER offer smaller memory footprints and faster inference speeds. Notably, CasMiner also possesses the unique advantage of enabling Cas9 identification without relying on a pre-specified query sequence. However, filtering based on Cas9-related keywords and Pfam domains to narrow the candidate pool, followed by applying CasMiner, proves to be an effective measure for mining novel Cas9 proteins. Consequently, CasMiner serves as a powerful complementary approach to existing homology search methods.

Finally, in this study, the editing efficiency of the Cas9 tools tested varied across different species. In a cross-species comparison, the highest efficiency was observed in rice, followed by HEK293T cells and maize. When compared within each species, VpCas9 exhibited comparable efficiency to SpCas9 in both rice and maize, with the mutant variants showing even higher activity. However, at certain target sites in mammalian HEK293T cells, SpCas9 performed better. These differences in editing efficiency, both across species for the same tool and across tools within the same species, may be attributed to factors such as the intracellular expression levels, target-site accessibility and species-specific DNA-repair mechanisms [50]. More systematic investigation and experimental validation will be required in the future.

## CONCLUSIONS

In this study, we developed CasMiner—a deep-learning model for the mining, design and optimization of Cas9 nucleases. CasMiner achieved a prediction accuracy of up to 99.63% and demonstrated strong generalization performance compared with other benchmark methods. As a proof-of-principle application, CasMiner was used to identify the functional nuclease VpCas9 and engineered three enhanced mutants (VPM2-1, VPM2-2 and VPM2-3). Experimental validation demonstrated that VpCas9 and its mutants achieved successful genome editing in rice, maize and HEK293T cells. Notably, in both rice and maize, the mutants VPM2-2 and VPM2-3 exhibited higher average editing efficiencies than SpCas9.

## MATERIALS AND METHODS

Detailed materials and methods are available as a Supplementary file.

## Supplementary Material

nwag090_Supplemental_Files

## Data Availability

The code, models for CasMiner and Supplementary Tables are open-sourced at Github: https://github.com/BRITian/CasMiner.
