# Supplementary material for: CasMiner: a deep-learning tool for high-throughput mining and rational design of efficient Cas9
Source: Natl Sci Rev. 2026 Feb 9;13(6):nwag090. doi: 10.1093/nsr/nwag090 (PMC13020425; doi:10.1093/nsr/nwag090)
Supplement: nwag090_Supplemental_Files [file nwag090_supplemental_files.zip › CasMiner-NSR__Supplementary-materials.docx]

# Supplementary Result

**1. Evaluation of the Edited Callus Rate**

Additionally, we also used our sequencing data to determine the proportion of individual tissue replicates that were successfully edited (i.e., the edited callus rate) as an alternative metric for assessing the editing efficiency of VpCas9 and its mutants. The results showed that all three mutants exhibited comparable or higher edited callus rates than wild type at most target sites. Specifically, excluding the four editing sites associated with OsDWF4 and OsGhd8, VpCas9 showed >59% edited callus rate. In contrast, the VPM2-2 mutant increased the VpCas9 edited callus rate of OsDWF4-FS0769 and OsDWF4-RS0770 from 21.9 % and 34.4 % to 78.6 % and 91.7 %, respectively. Taking the average of edited callus rates across the 12 target sites revealed that mutants consistently showed higher positive rates than wild type (VPM2-1, 65.48 %; VPM2-2, 67.95 %; VPM2-3, 68.80 %; VPCas9, 62.87 %; Figure S14A, Table S10). Taken together, these results indicated that VpCas9 mutants developed here presented markedly improved performance in rice genome editing.

**2. Generalization capability evaluations against other models trained on artificial sequences**

Initially, large-scale language models (LLMs), such as MP-TRANS and ESM2, ESM2-encoded CNN and LSTM models, as well as newly developed machine learning models based on Random Forest (RF) or Support Vector Machine (SVM), were compared with CasMiner. The evaluation results showed that all six models achieved high accuracy in predicting Cas9 sequences within the training dataset (Figure S18A, See Supplementary Method 24 for Training Methods).

To further assess the models’ abilities to discriminate authentic proteins from unrealistic, random sequences, all five independent test datasets (Cas9-100, Cas12-13, nuclease, helicase and glycoside hydrolases) underwent the same level of internal sequence shuffling as the training datasets (80%; these datasets are marked with an asterisk), resulting in a total of ten independent test datasets. The evaluation results are shown that CasMiner achieved a high prediction accuracy for authentic Cas9 proteins from the UniRef100 dataset (Cas9-100). It also demonstrated strong performance in distinguishing non-Cas9 sequences (authentic Cas12-13, nucleases, helicases, and glycoside hydrolases as well as unrealistic sequences, Figure S18B-C, Table S19). To be precise, only CasMiner (AUROC=1.0, AUPRC=1.0) could consistently distinguish Cas9 from non-Cas9 sequences (Figure S18D) and it achieved 100% Accuracy and Recall on the Cas9-100 dataset (Label = 1), and 100% Accuracy with 0% Recall on the other nine negative datasets (Label = 0) (Figure S19). The results further confirm that CasMiner’s generalization ability is superior to that of other models.

**3. Performance assessments relative to models built on authentic sequences**

We further collected real-world non-Cas9 sequences (ranging from 801 to 1,820 amino acids) from the SwissProt database as negative data. From this source, we constructed two negative datasets, a size-balanced dataset (1,946 randomly selected sequences, denoted as Swiss-equal (SE)) and a full dataset (32,487 sequences, denoted as Swiss-all (SA)). Subsequently, we developed six model variants: CasMiner’s framework (CNN-LSTM), and fine-tuned versions of ESM2-8M and MP-TRANS. The same evaluation matrix was performed and the result showed that all models achieved well performance on the training datasets (Figure S20A). However, the models constructed using these two real-world negative datasets exhibited significantly poorer generalization capabilities on both Cas12-13 proteins and internally sequence-shuffled Cas9 proteins. Notably, only CasMiner attained 100% in both accuracy and recall, outperforming all other models (Figure S20B–D, Table S20).

**4. Large-scale evaluations of CasMiner and other artificial-sequence-based models on the Swiss-all negative (non-Cas9) dataset**

Additionally, we further utilized this Swiss-all negative dataset, rich in both bacterial and non-bacterial proteins, to evaluate models relevant to synthetic sequence. The results showed that CasMiner achieved the highest prediction accuracies of 100% (bacterial protein subset) and 99.94% (non-bacterial protein subset), markedly outperforming SVM (the second-best performer in terms of generalization, with accuracies of 87.19% and 84.20% for the same subsets) as well as other AI-based models. (Figure S21, Table S21).

**5. The evaluation of computational resource consumption and runtime across different tools**

From the perspective of computational cost, since Cas9 proteins consist of long sequences, Transformer-based model would produce excessively large attention matrices, leading to prohibitive memory usage and time costs. For the independent test set of 5,484 sequences, ESM2_CNN-LSTM, ESM2_FT, and MP-TRANS_FT required 59 minutes with 77.2 GB, 17.4 minutes with 13.6 GB, and 17.5 minutes with 26.7 GB, respectively. In comparison, CasMiner, BlastP and HMMER offer a smaller memory footprint and faster inference speeds. Although CasMiner is comparable to or marginally less efficient than BlastP (4.6 minutes, 215.51 MB) and HMMER (15 seconds, 9.0 MB) in computational performance, it offers greater ease of use (Figure S23, Table S22). This advantage stems from its ability to perform searches without requiring query sequences.

# Supplementary Methods

**1. Collection of positive data set and construction of negative data sets**

A total of 2,173 Cas9-related sequences were retrieved from the UniRef90 data set via the UniProt web service using the query term “CRISPR-associated endonuclease Cas9”. Following length screening of the retrieved sequences, 1,947 core Cas9 sequences with lengths between 801 ($AVE(SEQ\_LEN)-STD(SEQ\_LEN) = 801.65$) and 1,820 amino acids ($\mathrm{MAX}\left( \mathrm{SEQ}_{\mathrm{LEN}} \right) = 1,816$) were obtained and used as a positive dataset. To construct negative data sets with varying disruption levels, we computationally disrupted the amino acid composition of each sequence (random seed = 1), applying disruption percentages from 10% to 100% in increments of 10%. This process generated 10 distinct negative datasets.

**2. Data coding strategy and model framework**

We used the One-hot coding method to encode 20 amino acids, so the protein sequence (SEQ) was converted by a 21 × 21matrix. For example, A (Ala, Alanine) was encoded as a vector ${(1, 0, 0, \cdots, 0, 0, 0)}_{(1 \times21)}$, Y (Tyr, Tyrosine) was encoded as a vector ${(0, 0, 0, \cdots, 0, 0, 1)}_{(1 \times21)}$, and nonstandard amino acids (nsAA) were encoded as zero vectors of $(1 \times21)$:

$$SEQ = \left( a_{n\_1}, a_{n\_2}, a_{n\_3}, \cdots,a_{n\_SEQ-LEN} \right)$$

$$a\in\left\{ \left[ \begin{matrix} A \\ C \\ \begin{matrix} \vdots\\ Y \\ \mathrm{nsAA} \end{matrix} \end{matrix} \right]_{(21 \times1)} = \left[ \begin{matrix} \begin{matrix} 1 & 0 \\ 0 & 1 \end{matrix} & \cdots& \begin{matrix} 0 & 0 \\ 0 & 0 \end{matrix} \\ \vdots& \ddots& \vdots\\ \begin{matrix} 0 & 0 \\ 0 & 0 \end{matrix} & \cdots& \begin{matrix} 0 & 1 \\ 0 & 0 \end{matrix} \end{matrix} \right]_{(21 \times21)} \right.$$

$$n\in(A, C, \cdots, W, Y,nsAA)$$

The model architecture started with an embedding layer, followed by two convolutional, maximum pooling layers, a Long Short-Term Memory (LSTM) layer, and a final prediction layer. In addition to the prediction layer, each layer was followed again by a Batch Normalization layer and a dropout layer. In this model, the rectified linear unit (ReLU) activation function was used (except for the final prediction layer) as follows:

$$ReLU\left( x \right) = \left\{ \begin{matrix} 0 \\ x \end{matrix} \right.\begin{matrix} , if x < 0 \\ , else \end{matrix}$$

where $x$ denotes the feature map from the convolution operation (the weighted sum of a neuron). The sigmoid activation function was used in the final prediction layer. We also verified the performance of the proposed method by a 10-fold cross-validation method. The detailed architecture and optimized hyperparameters are shown in Figure S1 and the DNN model were trained on the TensorFlow platform based on Keras 2.1.5 in the Python 2.7.15 programming environment. The code for the above DNN architecture is available from GitHub (https://github.com/BRITian/CasMiner).

**3. Evaluation of prediction performance**

A 10-fold cross-validation strategy was used to train and evaluate the performance of the prediction models. The performance of the prediction models was evaluated with metrics including accuracy, recall, precision, F1-score, and the area under the receiver operating characteristic curve (AUROC, AUC), which were each calculated based on 10-fold cross-validation. The AUC of the prediction model was greater than 0.5, indicating that the performance of the constructed models was better than a random classifier. The metrics were calculated using the Keras package as follows:

$$Recall = \frac{TP}{TP+FN}(0 \leq Recall \leq1)$$

$$Precision = \frac{TP}{TN+FP}(0 \leq Precision \leq1)$$

$$F1 = \frac{Precision \times Recall}{Precision+Recall}(0 \leq F1 \leq1)$$

$$Accuracy = \frac{TP+TN}{TP+TN+FP+FN}(0 \leq Accuracy \leq1)$$

where TP, TN, FP, and FN indicate true positive, true negative, false positive, and false negative, respectively.

**4. Collection of data sets for evaluation of model generalizability**

To evaluate the generalizability of the models, four distinct evaluation data sets were compiled from UniProt:

Helicase and Nuclease Dataset: Protein sequences annotated with “helicase” or “nuclease” were retrieved from the Swiss-Prot section of UniProt. Sequences were filtered to exclude any containing “CAS9”, “CSN1”, “CAS12”, “CPF1”, “CAS13”, or “C2C2” in their names and restricted to a length range of 801 to 1820 amino acids.

Cas12-13 Variants Dataset: Separate searches in UniProtKB were performed using the specific keywords “Cas12a”, “Cas12b”, “Cas12c”, “Cas12d”, “Cas12k”, “Cas13a”, “Cas13b”, “Cas13c”, and “Cas13d”. The resulting sequences were combined and filtered to exclude entries with “CAS9” or “CSN1” in their names and constrained to the same 801–1820 amino acid length range.

GH-UPKB Dataset: Sequences were retrieved from UniProtKB using the specific keyword 'glycoside hydrolase', followed by filtering for reviewed sequences and restriction to the same amino acid length range of 801–1820.

UniRef100 Cas9 (Cas9-100) Dataset: A larger set of Cas9 sequences was obtained by searching UniRef100 for “CRISPR-associated endonuclease Cas9”. This data set underwent filtering to remove sequences containing “CAS12”, “CPF1”, “CAS13”, or “C2C2” in their names and sequences shorter than 801 amino acids or longer than 1820 amino acids. Crucially, to prevent evaluation on training data, all Cas9 sequences used in model training were explicitly removed from this evaluation set.

**5. Model prediction and sequence characteristic extraction**

Model prediction and characteristic extraction were performed using the CasMiner prediction script (available at: https://github.com/BRITian/CasMiner/). For basic prediction, sequences were processed using the command: python CasMiner-Pred-V7.py /path/to/input.fa p80 0 1.

Simultaneous prediction and extraction of sequence characteristics were performed using the following command: python CasMiner-Pred-V7.py /path/to/input.fa p80 1 1.

**6. CRISPR analysis and the search for TracRNA**

The Taxonomy IDs (taxids) of the species were used to download the reference genomes through ncbi-genome-download 0.3.3 (https://github.com/kblin/ncbi-genome-download), and the CRISPR Recognition Tool (CRT) was used to determine Repeat and Spacer in the genomes ^1^. Then, BlastN was used to locate the TracRNA in the genome, where Expectation value (e-value) was set to ${10}^{-5}$, Word size for wordfinder algorithm (word_size) was set to 7, Penalty for a nucleotide mismatch (-penalty) was set to −2, Reward for a nucleotide match (-reward) was set to 1, Cost to open a gap (-gapopen) was set to 1, and Cost to extend a gap (gapextend) was set to 2. Finally, sgRNA was assembled by Repeat and TracrRNA.

**7. Protein expression and purification**

After determining the appropriate Cas9 sequence, the sequence was optimized by General Biosystems (Anhui) Co., Ltd (Anhui, China) according to the codon preference of *Escherichia coli* and inserted into the pET-28a plasmid. Subsequently, the pET-28a-Cas9 recombinant plasmid was transformed into *E. coli* BL21 competent cells. Subsequently, the *E. coli* BL21 monoclonal cells carrying the pET-28a-Cas9 recombinant plasmid containing the target gene were cultured in 50 mL of Lysogeny Broth (LB) medium containing kanamycin (50 μg/mL) at 37°C with shaking at 200 rpm until the optical density at 600 nm (OD_600_) was between 0.6 and 0.8, and then 20 mM isopropyl β-d-1-thiogalactopyranoside (IPTG) was added. Subsequently, the bacterial culture was placed at 16°C with shaking at 200 rpm for induction of protein expression for 18 h. The cells were collected by centrifugation at 8,000 rpm for 10 min, and resuspended in 8 mL of 20 mM phosphate buffer (PB, pH 7.0). The cells were lysed by ultrasonication at a power of 35 W using 4-s pulses with 4-s pauses, for a total of 10 min on ice-water. The samples were centrifuged at 8,000 rpm at 4°C for 30 min and the supernatant was obtained. After the imidazole 40 mM/L (NTA40) eluent was filtered by nickel column affinity chromatography, the imidazole 200 mM/L (NTA200) eluent was collected. The collected eluent was subjected to dialysis overnight and concentration with polyethylene glycol 8000, and the purified concentrated protein was quantified using a BCA protein quantification kit (Tiangen Biotech (Beijing)).

**8. Determination of PAM preference of Cas9**

The 150-bp fragment of PAM with 5′-NNNN-3′ ^2^ was synthesized by General Biosystems (Anhui) (**Supplementary Material 3**), and the corresponding sgRNAs were synthesized by Nanjing GenScript Biotechnology (Nanjing, China). The Cas9 protein, sgRNAs, and PAM library were diluted to 100 ng/μL, 2 pmol/μL, and 200 ng/μL, respectively. The RNP complex was formed by 8 pmol sgRNA and 300 ng Cas9 protein in the reaction solution containing Mg^2+^ at 37°C for 10 min. Then, 200 ng of PAM library fragments were added to the reaction at 37°C for 20 min. The reaction was terminated by adding a solution of 0.5% SDS and 150 mmol/L EDTA (pH 8.0). Finally, the reaction system was subjected to next-generation sequencing (NGS) (Shanghai Majorbio Bio-Pharm Technology, Shanghai, China).

**9. *In* *vitro* nucleic acid fragment cutting experiment**

The nucleic acid fragment with a length of 1657 bp was amplified using the designed primers (**Supplementary Material 4**), and 200 ng of the nucleic acid fragment was added to the RNP system and allowed to react at 37°C for 20 min. Finally, electrophoresis was performed, and the cleavage results of Cas9 were observed and recorded.

**10. *In* *vivo* growth fluorescence reporting system experiment**

The red fluorescent protein (*mApple*) gene was inserted into the *lacZ* gene of *E.* *coli* by multicopy chromosomal integration using CRISPR-associated transposase (MUCICAT) technology ^3^. The resulting strain, BMLacZ, retained the *mApple* target sitewas obtained after loss of the associated plasmid (**Supplementary Material 5**). Subsequently, 100 ng of pET-28a-Cas9 and 100 ng of pACYC-Duet-sgRNA recombinant plasmids were mixed and transformed into BMLacZ competent cells prepared by the calcium chloride (CaCl_2_) method. They were plated on LB medium plates containing kanamycin (50 μg/mL) and chloramphenicol (50 μg/mL), and cultured for 12 h at 37°C in an incubator.

The cells were inoculated into the wells of 96-well plates containing 200 μL of liquid LB with kanamycin (50 μg/mL), chloramphenicol (50 μg/mL), and IPTG (2 mmol/mL). The plates were then cultured at 37°C with shaking at 600 rpm for 8 h, and the OD_600_ and red fluorescence with OD_568_–OD_592_ were measured every hour.

**11. Design strategy of mutants**

The protein sequence of VpCas9 was submitted to jackhmmer, and the homologous sequences of VpCas9 were retrieved from the UniRef90 database. CasMiner’s Grad-CAM was then used to extract the characteristics of homologous sequences, and the characteristic matrix was summed and divided by the number of homologous sequences as a characteristic matrix to assist mutation of VpCas9. At the same time, the conserved (functional) matrix of VpCas9 was obtained by position-specific amino acid probabilities (PSAP). The score difference (Diff) between the optimal mutant and the wild-type at each site in the characteristic matrix and the conserved (functional) matrix was calculated. Under conditions to ensure consistency of the optimal mutants at the same point, the top 12 mutation sites (sum score < 30) were selected as candidate mutation points by sorting and adding the two Diffs.

**12. Construction of mutants**

A two-step PCR mutagenesis strategy was used to construct a single point mutation. First, the primers for mutation (**Supplementary Table 23**) were designed using snapgene. Subsequently, the wild-type plasmid was used as a DNA template and under the action of Phanta Max ultra-fidelity DNA polymerase (Nanjing Vazyme Biotech, Nanjing, China), the mutant fragment was amplified with T7-F primers and downstream primers containing the mutant sequence or T7-R primers and upstream primers containing the mutant sequence. PCR was performed with an initial denaturation step at 95°C for 5 min followed by 32 cycles of 95°C for 30 s, 58°C for 30 s, and 72°C for 2 min, and a final extension step at 72°C for 10 min. The target gene fragment was recovered using a DNA gel extraction kit (Nanjing Vazyme Biotech, Nanjing, China), and then used as primer to perform a second round of PCR amplification with the wild-type plasmid as a template. PCR was performed with an initial denaturation step at 95°C for 5 min followed by 32 cycles of 95°C for 30 s, 70°C for 30 s, and 72°C for 5 min, and a final extension step at 72°C for 10 min. After amplification, the wild-type plasmid was eliminated by digestion with *Dpn*I (New England Biolabs, Ipswich, MA, USA), and the mutant plasmid was recovered using a purification and recovery kit (Tiangen Biotech (Beijing)), and transformed into *E. coli* Top10 competent cells (Tiangen Biotech (Beijing)), by standard procedures. Then, monoclonal positive identification of the transformants was carried out with universal primers of T7-F and T7-R, and sequencing was performed by Beijing Tsingke Biotech (Beijing, China). Finally, the sequencing results were analyzed to determine the monoclonal containing the correct mutant. One mutation site can be added at a time with the above method, and the recombinant plasmid with multiple point mutations can be obtained by repeated operation.

**13. qPCR detection of genome editing activity**

The BMLacZ strain was cultured overnight, and its genome was extracted using a bacterial genomic DNA extraction kit (Tiangen Biotech (Beijing)). Subsequently, 2500 ng of BMLacZ genomic DNA was added to the RNP system, and the system was filled with sterile water to 20 μL for digestion at 37°C for 20 min. After 1:125 dilution, 1 μL of the digestion system was added to the qPCR reaction system (Nanjing Vazyme Biotech, **Supplementary Table 24**). Finally, the reaction and fluorescence detection were performed using a real-time fluorescence quantitative PCR instrument (Thermo Fisher Scientific (China), Shanghai, China).

**14. Molecular dynamics simulation and analysis**

The tertiary structures of SpCas9, VpCas9, and VpCas9 mutants were predicted using the AlphaFold 3.0 web service with the default parameters.

Subsequently, OpenMM molecular dynamics simulation was performed using the obtained structure files. The proteins were placed in a water box at pH 7.0, and then simulated at amber14-all.xml, amber14/tip3pfb.xml force field, and 310K temperature conditions for 2 fs per frame. A total of 250,000,000 steps were simulated, involving a duration of 500 ns, and a record was set every 25,000 steps. A total of 10,000 frames of simulation results were recorded, and the procedure was repeated five times for molecular dynamics simulation of each protein.

The standard deviation was calculated according to the root mean square deviation (RMSD), and the three data sets with the smallest standard deviation of each protein in the simulation were selected. Based on the best three data sets, RMSD, root mean square gluctuation (RMSF), principal component analysis (PCA), and free energy landscape (FEL) were further analyzed.

**15. Analysis of electrostatic potential of protein**

ChimeraX was used to analyze the electrostatic potential (ESP). The protein tertiary structure was imported into the software, and then the electrostatic potential was analyzed with the command “coulic protein key true” in the console.

**16. Construction of rice genome editing vector**

The vectors were constructed according to Xie’s methods, using the modified pRGEB32 vector ^4^. VpCas9, VPM2-1, VPM2-2, and VPM2-3 sequences were optimized for rice codons and synthesized by Nanjing GenScript Biotechnology. All four sequences were flanked with 4*NLS at both the5′- and 3′-ends of the coding sequence. For the backbone construction of VpCas9, specific primers were synthesized to amplify the above VpCas9. The PCR product was purified and cloned into the pRGEB32 (digested with *Snab* I and *Asc* I) with NEBuilder HiFi DNA Assembly Master Mix (New England Biolabs, Inc.), and designated as VpCas9-pRGEB. The tRNA followed by two *Bas* I restriction sites and Vp-sgRNA were synthesized by Nanjing GenScript Biotechnology and used to replace the original tRNA-sgRNA component in the pRGEB32 vector to generate the VpCas9-Vp-sgRNA backbone. Four target sites using tRNA-Vp-sgRNA architectures were designed and ligated using *Bsa* I according to Xie’s methods. VPM2-1, VPM2-2, and VPM2-3 were constructed according to the method described above. All primers are listed in **Supplementary Table 25**.

**17. Construction of maize genome editing vector**

The p62SK vector was digested with the restriction endonucleases *Sac*Ⅰ and *BamH*Ⅰ, after which the maize codon-optimized VpCas9, SpCas9, and VpCas9 mutants (synthesized by General Biosystems (Anhui) Corp. Ltd.) were individually inserted into the linearized vector. Subsequently, the *mCherry* targeting sites were designed using the online tool CRISPR-P ^5^, and the target expression cassettes (driven by the *Zm*U6 promoter and harboring the target sgRNA sequences) were synthesized by SynBio-Tech Co., Ltd. The p62SK-Cas9 vectors were then double-digested with either *Xba*Ⅰ/*SnaB*Ⅰ or *SnaB*Ⅰ/*Not*Ⅰ, and the two target expression cassettes were inserted into the vectors via restriction digestion and ligation. All ligation products were transformed into Escherichia coli DH5α competent cells. Finally, five endotoxin-free knockout vectors required for the experiment were extracted using the EndoFree Plasmid Midi Kit (Jiangsu CoWin Biotech Co., Ltd).

**18. Construction of HEK293T genome editing vector**

Based on the pSpCas9(BB)-2A-Puro (PX459) V2.0 vector constructed by Ran et al. ^6^, we optimized VpCas9 and its three derivatives with human codons. The optimized sequences were synthesized by General Biosystems (Anhui) Corp. Ltd., which were then used to replace the SpCas9 sequence in the original vector. Finally, transfection-grade plasmids were extracted and provided by the company.

**19. Agrobacterium transformation of rice callus cells**

The rice cultivar Nipponbare (*Oryza sativa* L. *japonica* cv. Nipponbare) was used in this study. All binary vectors were introduced into *Agrobacterium tumefaciens* strain EHA105 using the freeze/thaw method. Rice embryogenic calli were then infected with Agrobacterium as described previously ^7^. The incubated and recovered calli were selected on medium containing 50 mg/L hygromycin for 4 weeks to obtain resistant calli. Vigorously growing calli were then transferred to regeneration medium to generate green plants.

**20. Maize protoplast transformation**

For maize protoplast preparation, the middle segments of the second and third true leaves were excised from etiolated maize seedlings and cut into thin strips approximately 1 mm in width, which served as the material for protoplast isolation. Maize protoplasts were then prepared with reference to the method described by Zhu et al ^8^. Subsequently, 20 μg of endotoxin-free plasmids was mixed thoroughly with 200 μL of protoplasts. A total of 220 μL of PEG-Ca^2+^ solution was added to the reaction mixture, which was gently mixed again and incubated at room temperature (25℃) in the dark for 30 minutes to mediate plasmid transformation.

**21. HEK293T cell transfection**

HEK293T cells were cultured in DMEM medium (Gibco) supplemented with 10% fetal bovine serum (FBS, Gibco) and 1% penicillin-streptomycin (Gibco). The cells were maintained in a cell incubator at 37°C with 5% CO_2_. To achieve high transfection efficiency, passage 15 HEK293T cells were selected for subsequent experiments. The selected HEK293T cells were seeded into 12-well plates and cultured for 24 hours until the cell confluency reached 70%–80%. Transfection was performed using 2 μg of plasmids and 4 μL of Lipo293Tplus (Beyotime), and the cells were cultured for another 72 hours after transfection. Finally, 100 μL of 0.25% Trypsin-EDTA was added to each well to digest the cells. After neutralizing the trypsin with 2 volumes of complete growth medium, the transfected cells were centrifuged and collected.

**22. DNA extraction and next-generation sequencing**

Genomic DNA was extracted from transgenic calli and plants of rice and maize using the DNA quick Plant System (Tiangen Biotech (Beijing)). Genomic DNA from HEK293T cells was extracted using 200 μL Lysis Buffer for Direct PCR (Mouse Tissue) （Genesand Biotech) and 4 μL Protease K. For mutation frequency detection in rice calli, about 30 hygromycin-resistant calli were selected for DNA extraction for each construct. And three biological replicates were established for both maize and HEK293T cells. Target sequences were amplified using specific primers (rice: **Supplementary Table 26** **and 28**; maize: **Supplementary Table 30**; HEK293T cells: **Supplementary Table 31**) and the PCR products were subjected to NGS sequencing (rice: Beijing Tsingke Biotech, Fast NGS; maize: Sangon Biotech (Shanghai), PE150; HEK293T cells: Sangon Biotech (Shanghai), PE300) to determine the mutation frequency of each construct. Alignment of amplicon sequences to a reference sequence was performed using CRISPResso2 ^9^ (for rice and HEK293T cells) and CRISPR-GRANT ^10^ (for maize, to analyze on-target editing and fragment deletion events). During data analysis, total mutation rates were lower than 10% and editing types with fewer than 50 reads were filtered out.

**23. Off-target detection**

Potential off-target sites in rice were predicted using the offTarget tool on the CRISPR-GE web service (http://skl.scau.edu.cn/offtarget/)^11^. Meanwhile, potential off-target sites in maize and HEK293T cells were retrieved using the Cas-OFFinder ^12^ web service (http://www.rgenome.net/cas-offinder). The top 3–5 potential off-target sites with the highest scores based on different PAMs, as well as 10 off-target-prone sites corresponding to each target in maize and HEK293T cells, were selected for off-target detection. The sequences of the forward and reverse primers from 5′ to 3′ were: adapter sequence (for library construction and sequencing), tag sequence, and target-specific amplification primer sequence (**Supplementary Table 27,** **28, 30 and 31**). PCR products were obtained using 2× KeyPo Master Mix (Dye Plus) (Nanjing Vazyme Biotech). PCR products carrying different tag sequences were mixed and sent to Beijing Tsingke Biotech for Fast NGS sequencing (1000×). Meanwhile, PCR products for maize and HEK293T cells were generated using 2× Phanta Max Master Mix (Dye Plus) (Nanjing Vazyme Biotech) and sent to Sangon Biotech (Shanghai) for PE150 and PE300 sequencing, respectively. After obtaining the deep sequencing data, CRISPResso2 ^9^ was used for analysis, and statistical analyses were conducted using Python and Excel.

**24. Potential o Benchmark Training and Evaluation of Related Models**

All models subjected to detailed evaluation alongside CasMiner were trained on the same dataset (i.e., a positive Cas9 dataset and an 80% internally shuffled negative dataset). For the pre-trained models (ESM2-8M and MP-TRANS), their default architectures and fine-tuning procedures were adopted without extensive hyperparameter search. For classical machine learning models (Random Forest (RF) and Support Vector Machine (SVM)), rigorous hyperparameter optimization was performed using random search (200 iterations) combined with five-fold cross-validation on the training dataset. For CasMiner, systematic adjustment of the model’s key hyperparameters was conducted using the Hyperas package based on our previous network architecture ^13^.

**25. Species Origin Identification of SwissProt Sequences**

Protein sequences were downloaded from the SwissProt database. Subsequently, unique identifiers of the evolutionary lineage for each sequence were extracted based on the Organism cross-reference (Taxon ID, denoted as "OX") in the sequence name. TaxonKit ^14^ was then employed to retrieve the lineage information of each species, and the Kingdom level in the lineage was used to distinguish bacterial proteins from non-bacterial proteins.

**Reference**

1. Briner, A.E., Henriksen, E.D. & Barrangou, R. Prediction and Validation of Native and Engineered Cas9 Guide Sequences. *Cold Spring Harb Protoc.* (2016). doi:10.1101/pdb.prot086785

2. Chen, P. *et al.* A Cas12a ortholog with stringent PAM recognition followed by low off-target editing rates for genome editing. *Genome Biology* **21**, 78 (2020). doi:10.1186/s13059-020-01989-2

3. Zhang, Y.W. *et al.* Programming Cells by Multicopy Chromosomal Integration Using CRISPR-Associated Transposases. *Crispr J* **4**, 350-359 (2021). doi:10.1089/crispr.2021.0018

4. Xie, K.B., Minkenberg, B. & Yang, Y.N. Boosting CRISPR/Cas9 multiplex editing capability with the endogenous tRNA-processing system. *P Natl Acad Sci USA* **112**, 3570-3575 (2015). doi:10.1073/pnas.1420294112

5. Lei, Y. *et al.* CRISPR-P: A Web Tool for Synthetic Single-Guide RNA Design of CRISPR-System in Plants. *Mol Plant* **7**, 1494-1496 (2014). doi:10.1093/mp/ssu044

6. Ran, F.A. *et al.* Genome engineering using the CRISPR-Cas9 system. *Nat Protoc* **8**, 2281-2308 (2013). doi:10.1038/nprot.2013.143

7. Hiei, Y. & Komari, T. *Agrobacterium*-mediated transformation of rice using immature embryos or calli induced from mature seed. *Nat Protoc* **3**, 824-834 (2008). doi:10.1038/nprot.2008.46

8. Zhu, J.M. *et al.* Protoplast transient expression-based RNA-sequencing: A simple method to screen transcriptional regulation in plants. *Plant Physiol* **194**, 408-411 (2024). doi:10.1093/plphys/kiad495

9. Clement, K. *et al.* CRISPResso2 provides accurate and rapid genome editing sequence analysis. *Nature Biotechnology* **37**, 224-226 (2019). doi:10.1038/s41587-019-0032-3

10. Fu, H.C. *et al.* CRISPR-GRANT: a cross-platform graphical analysis tool for high-throughput CRISPR-based genome editing evaluation. *Bmc Bioinformatics* **24**, 219 (2023). doi:10.1186/s12859-023-05333-w

11. Xie, X.R. *et al.* CRISPR-GE: A Convenient Software Toolkit for CRISPR-Based Genome Editing. *Mol Plant* **10**, 1246-1249 (2017). doi:10.1016/j.molp.2017.06.004

12. Bae, S., Park, J. & Kim, J.S. Cas-OFFinder: a fast and versatile algorithm that searches for potential off-target sites of Cas9 RNA-guided endonucleases. *Bioinformatics* **30**, 1473-1475 (2014). doi:10.1093/bioinformatics/btu048

13. Ding, Z.D. *et al.* MPEPE, a predictive approach to improve protein expression in *E. coli* based on deep learning. *Comput Struct Biotec* **20**, 1142-1153 (2022). doi:10.1016/j.csbj.2022.02.030

14. Shen, W. & Ren, H. TaxonKit: A practical and efficient NCBI taxonomy toolkit. *J Genet Genomics* **48**, 844-850 (2021). doi:10.1016/j.jgg.2021.03.006

# Supplementary Materials

## Supplementary material 1: Sequence alignment results of SpCas9 and VpCas9

########################################

# Program: needle

# Rundate: Thu 16 May 2024 02:45:31

# Commandline: needle

# -asequence SpCas9-Prot.fa

# -bsequence VpCas9-Prot.fa

# Align_format: srspair

# Report_file: spcas9.needle

########################################

#=======================================

#

# Aligned_sequences: 2

# 1: SpCas9

# 2: VpCas9

# Matrix: EBLOSUM62

# Gap_penalty: 10.0

# Extend_penalty: 0.5

#

# Length: 1384

# Identity: 675/1384 (48.8%)

# Similarity: 940/1384 (67.9%)

# Gaps: 68/1384 ( 4.9%)

# Score: 3341.5

#

#=======================================

SpCas9 1 MDKKYSIGLDIGTNSVGWAVITDEYKVPSKKFKVLGNTDRHSIKKNLIGA 50

|...|:||||||||||||:||.::|::..:|..:.|:|...:.|||..|.

VpCas9 1 MRANYTIGLDIGTNSVGWSVIKEDYQLVRRKMPIYGDTTVKAQKKNFWGV 50

SpCas9 51 LLFDSGETAEATRLKRTARRRYTRRKNRICYLQEIFSNEMAKVDDSFFHR 100

.|||.|:|||:.|:|||.||||.||:||:.|||.||.:::.::|..||||

VpCas9 51 RLFDEGQTAESRRIKRTTRRRYLRRRNRLNYLQTIFKDDIHQLDAHFFHR 100

SpCas9 101 LEESFLVEEDKKHERHPIFGNIVDEVAYHEKYPTIYHLRKKLVDSTDKAD 150

||:||||::.|:|.::||||.:.:|:.||.:|||||||||:|.|||.|||

VpCas9 101 LEDSFLVKDAKRHTKYPIFGTLDEEINYHNEYPTIYHLRKELADSTQKAD 150

SpCas9 151 LRLIYLALAHMIKFRGHFLIEGDLNPDNSDVDKLFIQLVQTYNQLF---- 196

:||:|||:||::|:||||||||.||..|:.::..|.|.:..||:.|

VpCas9 151 IRLVYLAIAHIVKYRGHFLIEGQLNSANTSINNTFQQFLNNYNEKFITQV 200

SpCas9 197 ---EENPINASGVDAKAILSARLSKSRRLENLIAQLPGEKKNGLFGNLIA 243

..:|::.: |..::|::|:.|:||:.:.::|..|.||..|.|...|.

VpCas9 201 PGLALSPVDTT-VLVESIVTAKTSRSRKSDAILALFPSEKSTGTFAQFIK 249

SpCas9 244 LSLGLTPNFKSNFDLAEDAKLQLSKDTYDDDLDNLLAQIGDQYADLFLAA 293

|.:|...|||..|.|..|||||.||..|:::|..|||::||.:||:|:||

VpCas9 250 LIVGNQGNFKKTFALEADAKLQFSKIEYEEELGELLAEVGDDFADVFVAA 299

SpCas9 294 KNLSDAILLSDILRVNTEITKAPLSASMIKRYDEHHQDLTLLKALVRQQL 343

||:.|||.||.||......|.|.||||||:||.:|..||.|.|..||:.|

VpCas9 300 KNVYDAIELSGILSTKDTETNAKLSASMIERYTQHQNDLKLFKKYVREYL 349

SpCas9 344 PEKYKEIFFDQSKNGYAGYIDGGASQEEFYKFIKPILEKMDGTEELLVKL 393

|::|..||.|.||.||||||||.|::||||||:|..|:........:.|:

VpCas9 350 PKQYDTIFKDSSKKGYAGYIDGEATEEEFYKFVKKTLDNTPDAAYFIDKI 399

SpCas9 394 NREDLLRKQRTFDNGSIPHQIHLGELHAILRRQEDFYPFLKDNREKIEKI 443

::|:.||||||:|||.|||||||.||:|||..|..:|..::.|::||..|

VpCas9 400 DQENFLRKQRTYDNGVIPHQIHLDELNAILENQSKYYTSIEKNKDKIIDI 449

SpCas9 444 LTFRIPYYVGPLARGNSRFAWMTRKSEETITPWNFEEVVDKGASAQSFIE 493

:|||||||||||:..||.|.|:||||...|.||||.|.||...|:..|||

VpCas9 450 MTFRIPYYVGPLSNHNSSFGWLTRKSPGEIRPWNFTERVDTYQSSVDFIE 499

SpCas9 494 RMTNFDKNLPNEKVLPKHSLLYEYFTVYNELTKVKYVTEGMRKPAFLSGE 543

||||.|..||.||||||:|.||:.:.::|||||:.|..| ..:...|||.

VpCas9 500 RMTNNDSYLPTEKVLPKNSFLYQKYMIFNELTKLTYTNE-KNERLNLSGN 548

SpCas9 544 QKKAIVDLLFKTNRKVTVKQLKEDYFKKIECFDSVEISGVEDRFNASLGT 593

:|:.||:.|||.:|||| ::|.||||......||.:|.|:|..|||....

VpCas9 549 EKREIVNHLFKKHRKVT-RKLLEDYFANYYQLDSTQIDGIETAFNAKYTV 597

SpCas9 594 YHDLLKIIKDKDFLDNEENEDILEDIVLTLTLFEDREMIEERLKTYAHLF 643

|||.:|:...::|||:|.|.|.|||:|..||:||||:||.|:||.|...|

VpCas9 598 YHDFVKLGVPQEFLDDEANVDTLEDVVKLLTVFEDRKMIREQLKKYDTCF 647

SpCas9 644 DDKVMKQLKRRRYTGWGRLSRKLINGIRDKQSGKTILDFL-KSDGF---A 689

..:.:|:::||.||||||||:||:.|::||.:||:|:|:| :.||. .

VpCas9 648 SQETLKKMERRHYTGWGRLSQKLLVGLKDKTTGKSIMDYLIEDDGMPKNI 697

SpCas9 690 NRNFMQLIHDDSLTFKEDIQKAQVSGQGDSLHEHIANLAGSPAIKKGILQ 739

||||||||:|.||:|||||..||.:.|.:.:.:.|..|||||||||||||

VpCas9 698 NRNFMQLINDVSLSFKEDIAAAQPTVQEEDIRQVIHGLAGSPAIKKGILQ 747

SpCas9 740 TVKVVDELVKVMGRHKPENIVIEMARENQTTQKGQKNSRERMKRIEEGIK 789

::.:|:|:|.:|| |.|::|||||||.|:.::| :..|:|.:|:.:.

VpCas9 748 SLTIVNEIVGIMG-HPPQSIVIEMARSNEISKK----TNSRLKALEKMLA 792

SpCas9 790 ELGSQILKEHPVENTQLQNEKLYLYYLQNGRDMYVDQELDINRLSDYDVD 839

:..|.:|||:|..|.:|:|:||:|||||.|:|||...:|:|:.||:||||

VpCas9 793 DFQSDLLKEYPTSNDKLKNDKLFLYYLQAGKDMYTGADLNIHALSNYDVD 842

SpCas9 840 HIVPQSFLKDDSIDNKVLTRSDKNRGKSDNVPSEEVVKKMKNYWRQLLNA 889

||:||||:||||:||.||..|..||||||:|||.|:|||.|.:|::||:|

VpCas9 843 HIIPQSFIKDDSLDNCVLVSSKANRGKSDDVPSSEIVKKQKYFWKKLLDA 892

SpCas9 890 KLITQRKFDNLTKAERGGLSELDKAGFIKRQLVETRQITKHVAQILDSRM 939

|||::||:|||||:|||||:..||.|||:|||||||||||||||||||:.

VpCas9 893 KLISKRKYDNLTKSERGGLTPADKEGFIRRQLVETRQITKHVAQILDSQF 942

SpCas9 940 NTKYDENDKLIREVKVITLKSKLVSDFRKDFQFYKVREINNYHHAHDAYL 989

|...::| :.|:::||||.|.:.|||.|..||:||:|:|||||||||

VpCas9 943 NDGQEDN----QAVQIVTLKSSLTNQFRKQFNLYKIRELNDYHHAHDAYL 988

SpCas9 990 NAVVGTALIKKYPKLESEFVYGDYKVYDVR-KMIAKSEQEIGKATAKYFF 1038

|||||:.|::|||:|:.|||||.: |: ::|.:. |||.|...

VpCas9 989 NAVVGSLLLRKYPQLKPEFVYGKF----VKGRLINRF-----KATQKKDL 1029

SpCas9 1039 YSNIMNFFKTEITLANGEIRKRPLIETNGETGEIVWDKGRDFATVRKVLS 1088

|:|||.|.||:..:|:. .|||:|.. ....|:::.||

VpCas9 1030 YTNIMKFLKTDDRVADA-------------NGEIIWSP-TTIKTIKRTLS 1065

SpCas9 1089 MPQVNIVKKTEVQTGGFSKESILPKRNSDKLIARKKDWDPKKYGGFDSPT 1138

..|::||||.|.||...:.|:|.||.....||.||...|...||||:|||

VpCas9 1066 SKQMSIVKKVEKQTDKLTNETIYPKAAQGTLIPRKNGLDSTVYGGFNSPT 1115

SpCas9 1139 VAYSVLVVAKVEKGKSKKLKSVKELLGITIMERSSFEKNPIDFLEAKGYK 1188

:.||||: ...|||.|.| ..:::||:::::.::|.:.|.:||:.|:

VpCas9 1116 IVYSVLI--SHVKGKKKAL--TYDVIGISLLKQKTYEADKIAYLESLGF- 1160

SpCas9 1189 EVKKDLIIKLPKYSLFELENGRKRMLASAGELQKGNELALPSKYVNFLYL 1238

:...|..:|||:|||..|:|.||.||||.||||||:|.|..| .:.|

VpCas9 1161 -ISPTLHFELPKFSLFLQEDGTKRFLASADELQKGNQLVLSDK---MMAL 1206

SpCas9 1239 ASHYEK-LKGSPEDNEQKQLFVEQHKHYLDEIIEQISEFSKRVILADANL 1287

.:|.:| :.|:.:..: :|..|:...|.::..|..|:::.:.||..|

VpCas9 1207 IAHSKKAIAGNLDSLD----YVTTHREDYDRLLNDILVFARKYLAADNVL 1252

SpCas9 1288 DKVLSAYNKHRDKPIREQAENIIHLFTLTNLGAPAAFKYFDTTIDRKRYT 1337

.|:.:||..:|...|.|.||..|:|...|.:||...|.::|..|.|.||.

VpCas9 1253 KKIEAAYEINRQNTIIETAEAFINLLKFTKIGAAMEFNFYDCKIARHRYR 1302

SpCas9 1338 STKE---VLDATLIHQSITGLYETRIDLSQLGGD 1368

:..: :.|..:::||:|||||:|..:.:

VpCas9 1303 TKADFTAIFDGCVVNQSVTGLYESRWKIKR---- 1332

#---------------------------------------

#---------------------------------------

## Supplementary material 2: Partial genomic information of *Vagococcus penaei*

> Partial_genomic_information_of_Vagococcus penaei

……AAAAACCTAGAAGAAGTCACCGGTCAAGGGATTGAAGCTACTGTAAATGACCAAAGTGTACACGTTGGTAAAGCCAAATTCGCCGGTACTACTAATGAAGTTTCTGTTGACCAAACAGTTGTATATATTGCGATTAATAAAGAGTATGCGGGTTATATTACGTTTAAAGATACAGTACGACCAGAATCGAAAGCAACAATTCAACAACTTAATGAATTAGGTATTCATCAAACGGTGATGCTAACAGGTGATCATCAATCAATTGCCGAACAAATCGGTCGTCAAGTTGGTATTTCTGATATTCATGCTAACTGTTTGCCTGAAGAAAAAATCGAGGTACTTAAAAAATTAGAACAAACCAGTCGTCCAGTTATTATGGTAGGTGACGGGGTCAATGATGTATTAGCTCTAACTGTCGCTGATGTAGGGATTGCAATGGGAGCTACAGGATCAACTGCAGCTAGTGAGAGTGCTGATGCTGTTATTTTAAAGGATGATTTAACTCGGGTCGCAGAGGCTGTGCGACTATCTAAAGATACCATGCGTATTGCACGACAATCGGTTTTAATTGGATTGCTGATTTGTGTTGTTTTAATGCTAATTGCGAGTACTGGTGTCATTCCAGCATTGATTGGGGCTGTCCTACAAGAAGTTGTTGATACGGTATCCATTCTCTCCGCATTAAGAGCTCGTCGTGAGTTGCCACTGAAAGTAAAACATTAATATCTAAATATTATTAAGAAGTATTGACTATCTTTTAAGTAGATAGTTTTTTACATGAACTACAATTAGATTATAGTAAATTTTTATTTGATAATGACAAAAAGTCAAGAGCACTTACTTAGTAGTGTTCTTGACTTTTTATCATTATTTGTTATTTATATATATAAAATATGTTATAATTTAGGTAAATAAAAAAAGCACCGAGACAGTGCCACTAGAATAGTTGCTTACGGACTAAGCCTTATTTTAACTTGCTATGTTGTTTTGAATGCTACCAACAACGTTATTATAGCATATTGGAAAAATTATGCAAGAGTTGACTGCAATAAGAGTGCGGATAAATAGGAGGAGAAGTAGATGCGAGCGAATTATACAATTGGATTAGATATTGGAACAAATTCGGTGGGTTGGTCAGTGATAAAAGAAGATTACCAACTTGTTAGACGGAAAATGCCGATTTATGGAGATACGACAGTTAAGGCACAAAAGAAAAATTTTTGGGGAGTTCGTTTATTTGATGAAGGACAGACGGCGGAGAGTCGACGCATCAAAAGGACCACTCGTCGTCGCTATCTTAGAAGGCGTAACCGTTTAAACTATTTACAAACAATTTTCAAAGATGACATTCATCAACTAGATGCTCACTTTTTTCATCGACTAGAAGATAGTTTTTTAGTGAAAGATGCCAAACGCCACACTAAATATCCGATTTTTGGTACATTAGATGAGGAAATTAACTACCACAATGAGTATCCCACAATTTATCATTTGCGCAAAGAACTAGCTGATTCCACACAAAAAGCAGATATTCGATTAGTTTACTTAGCTATTGCCCATATCGTTAAATATCGAGGCCATTTTCTAATTGAAGGTCAGTTGAATTCAGCCAATACGTCAATTAATAATACATTCCAACAATTCTTAAACAATTACAATGAAAAATTTATTACGCAAGTTCCTGGATTAGCTTTGTCACCAGTTGATACTACTGTTTTAGTCGAGTCAATTGTTACAGCTAAGACCTCACGTAGTCGAAAAAGTGACGCAATATTGGCACTCTTTCCATCTGAAAAATCAACGGGTACGTTTGCCCAATTTATTAAATTAATTGTAGGGAATCAAGGGAATTTTAAAAAGACATTTGCTTTAGAGGCAGATGCCAAACTACAATTTTCCAAGATTGAATACGAAGAAGAACTGGGCGAACTCTTGGCAGAAGTAGGGGATGATTTTGCTGATGTGTTTGTTGCGGCAAAAAATGTATACGATGCCATCGAATTATCAGGCATATTAAGTACAAAAGATACAGAAACAAATGCAAAATTATCCGCGAGTATGATTGAACGTTATACCCAGCATCAGAACGATTTGAAATTGTTTAAGAAATATGTGCGTGAATATCTACCAAAACAGTACGATACTATCTTTAAGGATAGTAGCAAAAAAGGCTATGCTGGGTACATTGATGGTGAAGCAACAGAGGAAGAATTTTATAAATTTGTTAAAAAGACGTTAGACAATACGCCAGATGCGGCATATTTTATTGACAAAATTGACCAAGAAAACTTTTTACGAAAACAACGAACGTATGATAATGGCGTGATTCCTCATCAAATTCATTTGGATGAGCTAAATGCCATTCTTGAAAATCAATCAAAGTATTACACGTCAATTGAAAAAAATAAAGATAAGATAATAGACATTATGACATTTAGAATTCCTTATTATGTGGGTCCTTTGAGTAATCATAATAGTTCGTTTGGTTGGTTAACTAGAAAGTCCCCTGGTGAGATTAGACCATGGAATTTTACTGAACGTGTTGATACATACCAATCATCTGTTGATTTTATTGAAAGGATGACCAATAATGACAGTTACTTACCGACTGAAAAAGTCTTACCTAAAAATAGTTTCTTGTATCAGAAATACATGATTTTTAATGAGCTGACTAAACTGACGTATACTAATGAAAAAAATGAACGTCTCAACTTATCCGGCAATGAGAAACGAGAGATAGTCAATCATCTATTTAAAAAACATCGAAAAGTAACACGTAAATTGCTAGAAGATTATTTTGCTAATTATTACCAATTGGATAGCACACAAATTGATGGGATTGAAACCGCCTTTAATGCTAAATACACGGTGTATCATGACTTTGTAAAATTAGGTGTTCCACAAGAATTCCTTGATGATGAAGCCAATGTAGATACATTAGAAGATGTGGTGAAATTATTAACGGTTTTTGAAGACCGTAAAATGATCCGTGAACAACTAAAAAAATATGACACGTGCTTTAGCCAAGAGACATTGAAAAAAATGGAACGCCGTCATTATACTGGCTGGGGGAGACTGTCGCAAAAATTATTAGTTGGTTTAAAAGATAAGACTACTGGTAAGTCAATAATGGATTATTTGATTGAGGACGACGGTATGCCTAAAAATATCAATCGTAATTTCATGCAGTTGATTAATGATGTTTCTTTGTCTTTTAAAGAAGATATCGCAGCAGCACAACCAACAGTACAAGAAGAAGACATCCGTCAAGTTATTCATGGTTTAGCAGGAAGTCCAGCTATCAAAAAAGGCATTCTTCAAAGTTTGACTATCGTTAATGAAATTGTTGGTATCATGGGTCACCCGCCACAGTCAATTGTCATTGAGATGGCTAGGAGTAATGAAATCAGTAAAAAAACGAATTCTCGATTAAAAGCCTTAGAAAAAATGTTAGCAGATTTTCAAAGTGATTTATTGAAAGAGTACCCGACATCGAATGATAAATTAAAAAATGACAAGTTATTTTTATACTATCTACAGGCTGGAAAAGACATGTATACAGGTGCCGACTTAAATATTCATGCATTATCTAATTACGATGTGGATCACATTATTCCGCAAAGTTTTATTAAAGATGATTCATTAGATAATTGTGTGTTAGTGAGTTCAAAGGCAAACCGAGGAAAATCAGATGACGTCCCAAGTAGTGAAATTGTTAAAAAACAAAAATATTTTTGGAAAAAATTGTTAGATGCGAAACTTATTAGTAAGCGAAAATACGATAATTTGACGAAGAGCGAACGTGGGGGACTGACACCTGCGGATAAAGAGGGGTTTATTAGAAGACAATTGGTTGAAACAAGACAAATAACGAAACACGTTGCCCAAATTTTAGATAGTCAGTTTAATGACGGGCAGGAAGACAATCAAGCAGTACAAATTGTCACTTTGAAGTCCTCTCTGACTAACCAATTTAGAAAACAGTTTAATCTCTACAAGATTAGGGAATTGAATGACTATCATCATGCACATGATGCGTATTTGAATGCCGTCGTGGGGTCATTGCTTTTACGTAAGTATCCACAACTGAAACCAGAGTTTGTGTATGGTAAATTTGTGAAGGGCCGTTTGATTAATCGGTTCAAAGCGACACAAAAAAAAGACCTCTACACCAATATCATGAAGTTTTTAAAAACAGATGACCGAGTAGCCGATGCTAATGGAGAAATTATCTGGTCACCTACAACGATAAAAACGATTAAACGCACACTATCTTCTAAGCAAATGTCTATTGTCAAAAAAGTTGAGAAGCAAACGGACAAACTAACAAATGAAACCATCTATCCTAAAGCAGCTCAAGGGACGCTGATTCCACGTAAAAATGGGCTTGATTCGACCGTATATGGTGGTTTTAATAGTCCTACGATTGTTTATTCTGTCTTGATTTCACATGTAAAAGGTAAGAAGAAAGCTCTCACTTATGACGTGATAGGTATTTCATTATTGAAACAAAAAACCTATGAAGCTGATAAAATAGCATACCTAGAGTCATTAGGATTTATCTCACCAACGCTTCATTTTGAATTACCTAAATTTTCCTTGTTCTTACAAGAAGATGGGACGAAACGCTTCTTAGCTAGTGCCGATGAGTTACAAAAAGGCAACCAATTAGTTTTATCAGATAAGATGATGGCGTTGATTGCCCACTCTAAAAAAGCCATCGCCGGTAATTTAGATAGCTTAGACTATGTGACCACCCATCGAGAAGACTACGATCGATTACTTAATGACATATTAGTATTTGCTAGAAAGTATTTGGCAGCAGATAATGTACTGAAAAAAATTGAAGCGGCCTACGAAATAAATCGCCAGAATACTATCATTGAAACGGCTGAAGCCTTTATCAATTTACTAAAATTCACTAAAATAGGAGCGGCTATGGAATTTAATTTTTATGATTGCAAGATAGCTAGGCATAGATACCGAACTAAAGCGGATTTCACTGCTATTTTTGACGGATGTGTTGTCAATCAGTCAGTGACGGGACTTTATGAGTCACGTTGGAAAATTAAGAGGTAGCAATTATGGGATGGCGGACAGTGATTATCAACACACACTCGAAATTATCTTATCAAAATAATCACCTTATTTTTAAATCTGTTGACAGTCGAGAAATGATTCATTTATCGGAAATAGATAGTCTATTGTTAGAGACCACTAATATCACGATTACAACTATGTTGATGAAACGCTTAATTGACGAAAATATTTTAGTGATTTTTTGCGATGATAAGCGGTTGCCAATTGGTAAAATGATGCCGTTTTTTGGTTGTCACAATAGTAGTCTCCAGTTACCAAAACAAATTAATTGGTCGTATGAACAAAAAGCAACTATTTGGACAGACATTATTTCACAAAAAATCATTAATCAAAGTTACTTTTTAAAACACTTGGCTTTTTATGAGAAATCAGATGCGATTTTGTATTTACATGATTCGTTGGGTCTTTTTGACCCAACAAATCGTGAAGGTCATGCAGCGAGAATTTATTTTAATACGTTATTTGGTACTAAATTTACAAGAGAGTCAGAAAATGACATCAATGCCGGTTTAAATTATGGATATACACTATTGATGAGTTTGTTTGCTAGAGAAATTAGTAAAAATGGTTGTATGACACAAATTGGACTCAAACATGCCAACCAGTTTAATGATTTTAATTTGGCAAGTGATTTGATGGAACCATTTCGTGTCTTAGTGGATGCTATCATCTATGAAAATCGAGATGAACCATTCTATGTAATGAAACGATGTCTATTTGATTTATTCAATGACACGTATCATTACGATAATAAAGAAATGTTTTTAACTAATATTGTTAGTGATTATACAAAAAAAATAGTAAAAGCTTTAAATGGAGAAAG**GGAAGGAGTTCCTATGTTTAGGATATGA**GTTATCGATATATGAGAATGTTAGTTATGTTTGATATACCAACAGAAACGGCGGATGATAGAAAAGCCTATCGCTTATTTCGCAAATTTTTACTCAGTGAAGGGTTTATTATGCATCAATACTCTGTCTATAGCAAAATTTTATTAAATAATTCAGCGAGTCAAGCGATGACAGCACGATTGAAAAAGAATAATCCTAAAAAAGGCTTAATTACTGCGTTAACGGTGACTGAAAAACAATTTTCTAAAATGCTTTATTTACATGGAGAGTATGATACAACTGTTAGTAATACAGATACTAGAATTATTTTTTTAG**GAGATAATTATGAGACACATTAA**TTTCCCTATTTTAGATGAGCCACTCTCAATTAACAAGGCCACATTTTTAGTAGTAGAGGAGAGAGATTTATTTACAAGACTGATTCGTTTATTTTATCAGTATGAAGAAGCTGGAGAGCTGAAACTCTATAAACAGGATTACCAGTCCATCAATAAAAGTGAACTATTGGTTATTACGGATATCTTAGGTTTTGACATAAATGCAGCAAGCGTGTTAAAACTCATCTATTCTGATTTGGAACAACAATTAAATGAGTCACCAGATGTTAAGACACAGATTGAAGACTTGTCACTGGGGATTACGCGCCTAATTGAAGTAGAGTTACTTAATCATGAGTTAGATTTAGAACTGGACGATATTACTTTTTTAGAACTATTAAAAATCTTAGGCGTTAAGATTGAAACAAAAACAGACACACTATTTGAAAAAATGTTAGAAATTATTCAGGTTTTTAAGTACCTGTCAAAGAAAAAATTCATTGTTTTTATTAATGTCTGTTCGTATTTTAATCAAGAAGAATTAATTAAAATAAGTGAATATATTTCGTTATTTGATAGCGATGTTTTGTTTTTGGAACACTATAAAATTGAAGGCGTTAATCAATTTATTATCGACAAAGACTATTATGTTACAAGTGAAACTATGTTATAATAAACTTAAAGACTGTTCCTTGAAAAAAGAATAATCTACTAACTAAATTAGCATTCAAAAGAGACATCTTGCTATGGATGAATGGCGCGATTACGGAACCGAGAAATTTTCTGCGAGGTTTTAGAGCTATGCTGTTTTGAATGCTTCCAAAACTGCTTGCTATACAGTAGCCAAGTACCACTTGTTTTAGAGCTATGCTGTTTTGAATGCTTCCAAAACCAAGCAAAAATTTTGTAGTGCTAAATGTCAGTTTTAGAGCTATGCTGTTTTGAATGCTTCCAAAACCCTAGTTCTGTTGCGTTTAACCACTCCTTACGTTTTAGAGCTATGCTGTTTTGAATGCTTCCAAAACGTCGAGACTCGAAATGCTTAAAGCTAACATGTTTTAGAGCTATGCTGTTTTGAATGCTTCCAAAACACGAATATAAATAGATCGACTGTTGAGTACGTTTTAGAGCTATGCTGTTTTGAATGCTTCCAAAACTATTGATCACATTATTCCTGTAAGCAAAGGGTTTTAGAGCTATGCTGTTTTGAATGCTTCCAAAACAATTCGGTTACAGCAAGTTTTTGCTGAGAAGTTTTAGAGCTATGCTGTTTTGAATGCTTCCAAAACATTTAAACGCCATTCATAACCATCTCGTCTGTTTTAGAGCTATGCTGTTTTGAATGCTTCCAAAACATGGAGTGAAGCAATTCCCAGAGTTAGCTTGTTTTAGAGCTATGCTGTTTTGAATGCTTCCAAAACTCGTTGGACTTGGGGACTGATTATCACGATGTTTTAGAGCTATGCTGTTTTGAATGCTTCCAAAACAGAAAAACAACGAGTTAAAGATGTTTTAACGTTTTAGAGCTATGCTGTTTTGAATGCTTCCAAAACAAAAGATATTTAAGTCAAAGAAGCGCAGCGGTTTTAGAGCTATGCTGTTTTGAATGCTTCCAAAACTTGGAGAAACTACTATCGCTAAAGGAACGTGTTTTAGAGCTATGCTGTTTTGAATGCTTCCAAAACGATACCGTCCCAAGCACCTTTTAAAACTCCGTTTTAGAGCTATGCTGTTTTGAATGCTTCCAAAACATCGATAGTGCCGTCCGCATTAATTTTTAAGTTTTAGAGCTATGCTGTTTTGAATGCTTCCAAAACATTTGCTTGTCCGCCAAGACTATGAGCAAAGTTTTAGAGCTATGCTGTTTTGAATGCTTCCAAAACACGACTAAGTGAGCAAATACAAACGCTAGAGTTTTAGAGCTATGCTGTTTTGAATGCTTCCAAAACTGTCCTATGCGACTGGTGGTATGGTTATGTGTTTTAGAGCTATGCTGTTTTGAATGCTTCCAAAACTTACTCTTTTAGCAAACGGTAATTTTAAACGTTTTAGAGCTATGCTGTTTTGAATGCTTCCAAAACAGAGTCTCAATCAGCCATGAACGTACTAGTGTTTTAGAGCTATGCTGTTTTGAATGCTTCCAAAACGGAAGAGTTGGATTTATTCCAAGATTTTGAGTTTTAGAGCTATGCTGTTTTGAATGCTTCCAAAACTAACTATTGTAGCGTCAAATTTGGCTAACCGTTTTAGAGCTATGCTGTTTTGAATGCTTCCAAAACTATGCGTCGATTGAGATTAGCAGTTAATATAGTTTTAGAGCTATGCTGTTTTGAATGCTTCCAAAACACGCATTTAATGGTGAAGCTACAGGAGTTGGTTTTAGAGCTATGCTGTTTTGAATGCTTCCAAAACATGTGACCGAAAATGGTTACACGTTTAGTCGTTTTAGAGCTATGCTGTTTTGAATGCTTCCAAAACAACAAACACTAAACAATATCCAGCTCAATTGTTTTAGAGCTATGCTGTTTTGAATGCTTCCAAAACTGTTGTAACTCAATTTGAGCTGGTGCGTACGTTTTAGAGCTATGCTGTTTTGAATGCTTCCAAAACAGAGCATTTGGAATGGAAGCTGAGCAAGCAGTTTTAGAGCTATGCTGTTTTGAATGCTTCCAAAACATGTTGGCGAGGAAATAGTCGGCATTAACAGTTTTAGAGCTATGCTGTTTTGAATGCTTCCAAAACTTAACAGGAGCTACTAGAGCGACTAAAACAGGTTTTAGAGCTATGCTGTTTTGAATGCTTCCAAAACAAAGACGAGGAAACAGGTGCAGCAACTGGTGTTTTAGAGCTATGCTGTTTTGAATGCTTCCAAAACATCTATTTGAGTTATCAGTTAAAGAGAAGGGTTTTAGAGCTATGCTGTTTTGAATGCTTCCAAAACTTGAGCGTAAAGCTAAGATGATATCTCCGTGTTTTAGAGCTATGCTGTTTTGAATGCTTCCAAAACCGAATAAACTTCTAGACGAACAAAATACTAGTTTTAGAGCTATGCTGTTTTGAATGCTTCCAAAACTACTCGTCTTCGATTTCCCACACTTTAGCTGTTTTAGAGCTATGCTGTTTTGAATGCTTCCAAAACTCAATTCAAAAAAGGTGATGAACCTAGTTTGTTTTAGAGCTATGCTGTTTTGAATGCTTCCAAAACAAGGTGACGTGTGGTTTAAACGTTTGCCCGAGTTTTAGAGCTATGCTGTTTTGAATGCTTCCAAAACTGTGAGCTCTTAGGCACTCCTACACCATTTGTTTTAGAGCTATGCTGTTTTGAATGCTTCCAAAACGATTAATTGTCTAATGTTGTCATATTCTTGGTTTTAGAGCTATGCTGTTTTGAATGCTTCCAAAACTGTTAATCGAGCTAGTGAAGAAGCAACGCAGTTTTAGAGCTATGCTGTTTTGAATGCTTCCAAAACGCTAGCTTGCGCTTTGCTTGTGAACACTTTGTTTTAGAGCTATGCTGTTTTGAATGCTTCCAAAACGCATAAACTCTACGTTAGTGCTACGACATTTGTTTTAGAGCTATGCTGTTTTGAATGCTTCCAAAACCAGTTGTAGTTTTTAATTGATTCTCATAGCGTTTTAGAGCTATGCTGTTTTGAATGCTTCCAAAACTGCTACATAAGGTTTGTGTTTGCGACTTCCGTTTTAGAGCTATGCTGTTTTGAATGCTTCCAAAACCAACGAGATTGCAAGCATGAGTCGAAATGTGTTTTAGAGCTATGCTGTTTTGAATGCTTCCAAAACTAATCCAACCGCCTGGAGTTAAACCGACATGTTTTAGAGCTATGCTGTTTTGAATGCTTCCAAAACCGTTGGGGGAGTAAGGATAGATGATAGGGAGTTTTAGAGCTATGCTGTTTTGAATGCTTCCAAAACACGTAAATTGACTAAAGAGACAGAAGGTGCGTTTTAGAGCTATGCTGTTTTGAATGCTTCCAAAACTGTCTGCTCGAAAGTGCTAAATTCTGATTTGTTTTAGAGCTATGCTGTTTTGAATGCTTCCAAAACCGCTCCGTACGCTGTCATACCACGTACACTGTTTTAGAGCTATGCTGTTTTGAATGCTTCCAAAACTATTTTTCTACAAGTTTCTTCTGGGCATCCGTTTTAGAGCTATGCTGTTTTGAATGCTTCCAAAACAAACAATAACGTCTCAATCATTGCTGACAAGTTTTAGAGCTATGCTGTTTTGAATGCTTCCAAAACTTCTCGCCAATCTTCTAATCCTTCTATTTCGTTTTAGAGCTATGCTGTTTTGAATGCTTCCAAAACTAGCAAAGAAACAATCGAAACTATCAATATGTTTTAGAGCTATGCTGTTTTGAATGCTTCCAAAACCATCTTTTTAGGCTTTCTAATATCTGCGTCGTTTTAGAGCTATTGCTGTTTTGATTTATTTAAGATGATGAATGTGAGATTATTTTCACCTTCATTATCTTTTTTTGTTTATTTGTCTCATGCGGTTTGTTTTTCTTTATTAATTTTTTATTTCTCTGAATCTGATATCATTAAAAACAGTTCCTACTAGTTAAAGGGATAGAAAATGTAGTTCCTATAATAATTATGCTGTTTTTATTTTTAATTCATAATACTTTTTTATATGTTTAGTCATAACTTGTTGCTCTTTTTAGCTTTGACCTTTGTGGATAATTAAATTAAGACATAGTCTGTGTGTAAATAATTAAAAAAGGATTGATGAATCGAAAATTGATTCATCAATCCTTTAAATTATAAGTTCTTTTTTTATATATTATCTTCGCCACTGCTGTTTGCCATGTTGTTGCCTTGGTCTTGTACCGTCATAACGACAATTTTTTTGCCTGTTTTGACATGTGTTAGATTCTGACATTTGTGGTCTGCTATTGGGTTCAGAGTTAGTGTCTTGAAGTGGACAATCAGTTGGTTTATTTTCTGTTTGTTGTCCTTTTTTTTGTGTATCCATTGAAGAATCACGTTGGATTGGACGGTTAAATCGCTGATATTTTCTAGCAGGTTGATGCGTATTTGTTCCGTTAACTAGTTGACAGGAATTAAAATGTGAAAAACGTTGTGCTTCAGGTAAATGACAAATTGGACAATTTTCTCTTGAGATTTTTGTATCCGTTGCTTCCGCCAATGTTTCTCGATAGATACAGTTACCATCAAAAGGTTCAGCGCTTACAAAAATAGTTACCAAAGACGACAGACTAAATATTAGAGCAACACTCAAAAAAAATTGTTTTAAATACCTCGACAT……

Note: The green font is TracrRNA sequence, the fragment complementary to TracrRNA and crRNA has a cyan background, the red font is VpCas9 coding sequence, the Cas1 sequence has a yellow background, Cas2 sequence has a red background, Csn2 sequence has a purple background, and the crRNA sequence has a green background.

## Supplementary material 3: A 150 bp PAM library was designed, which contained a 20 bp sequence of the target and a random PAM sequence with 5'-NNNN-3'.

5'GGTGAAGAGAACAACATGGCTATTATTAAGGAGTTCATGC 40 bp

GTTTTAAGGTCCACATGGAGGGTTCCGTTAACGGTCATGA 80 bp

ATTTgaaattgagggtgagggtgaNNNNAGACCATACGAA 120 bp

GCTTTTCAAACTGCTAAGTTGAAGGTCACC 3' 150 bp

Note: There is a green background on the gRNA fragment and a cyan background on the PAM region.

## Supplementary material 4: The designed nucleic acid fragment with a length of 1,657 bp.

GGGCGCATCGTAACCGTGCATCTGCCAGTTTGAGGGGACGACGACAGTATCGGTGTTGATACAACCATAAAATGATAATTACACCCATAAATTGATAATTATCACACCCATAAATTGATATTGCCTCTTCATGGTCTAAACTTCAGTAAGTTTACGACATTTTCCTCGAGGTCATTTCCGGGGATCCATGGAGAAAAAAATCACTGGATATACCACCGTTGATATATCCCAATGGCATCGTAAAGAACATTTTGAGGCATTTCAGTCAGTTGCTCAATGTACCTATAACCAGACCGTTCAGCTGGATATTACGGCCTTTTTAAAGACCGTAAAGAAAAATAAGCACAAGTTTTATCCGGCCTTTATTCACATTCTTGCCCGCCTGATGAATGCTCATCCGGAGTTCCGTATGGCAATGAAAGACGGTGAGCTGGTGATATGGGATAGTGTTCACCCTTGTTACACCGTTTTCCATGAGCAAACTGAAACGTTTTCATCGCTCTGGAGTGAATACCACGACGATTTCCGGCAGTTTCTACACATATATTCGCAAGATGTGGCGTGTTACGGTGAAAACCTGGCCTATTTCCCTAAAGGGTTTATTGAGAATATGTTTTTCGTCTCAGCCAATCCCTGGGTGAGTTTCACCAGTTTTGATTTAAACGTGGCCAATATGGACAACTTCTTCGCCCCCGTTTTCACTATGGGCAAATATTATACGCAAGGCGACAAGGTGCTGATGCCGCTGGCGATTCAGGTTCATCATGCCGTTTGTGATGGCTTCCATGTCGGCAGAATGCTTAATGAATTACAACAGTACTGCGATGAGTGGCAGGGCGGGGCGTAATTTTTTTAAGGCAGTTATTGGTGCCCTTCTAGAGTCTTGACGGCTAGCTCAGTCCTAGGTACAGTGCTAGCTACTAGAGAAAGAGGAGAAATACTAGATGGTTTCTAAGGGTGAAGAGAACAACATGGCTATTATTAAGGAGTTCATGCGTTTTAAGGTCCACATGGAGGGTTCCGTTAACGGTCATGAATTTGAAATTGAGGGTGAGGGꜜTGAGGGTAGACCATACGAAGCTTTTCAAACTGCTAAGTTGAAGGTCACCAAGGGTGGTCCATTGCCATTTGCTTGGGATATTTTGTCTCCACAATTTATGTACGGTTCCAAGGTTTACATCAAGCACCCAGCTGATATTCCAGATTACTTTAAGTTGTCCTTCCCAGAGGGTTTTAGATGGGAAAGAGTTATGAACTTTGAGGACGGTGGTATTATCCACGTTAACCAAGATTCTTCCTTGCAGGATGGTGTTTTTATTTACAAGGTCAAGTTGCGTGGTACCAACTTTCCATCTGATGGTCCAGTTATGCAAAAGAAGACTATGGGTTGGGAAGCTTCTGAAGAAAGAATGTACCCAGAAGATGGTGCTTTGAAGTCTGAAATTAAGAAGCGTTTGAAGTTGAAGGACGGCGGTCATTACGCTGCTGAAGTTAAGACTACTTACAAGGCTAAGAAGCCAGTTCAATTGCCAGGTGCTTACATTGTTGATATTAAGTTGGACATCGTCTCCCACAACGAGGATTACACTATTGTTGAACAGTACGAGAGAGCCGAAGGTAGACATTCTACTGGTGGTATGGATGAATTGTACAAGTAAGC

Note: As shown in Figure S10D, the green font is the chloramphenicol resistance gene, the red font is the *mApple* gene, the yellow base fragment is the gRAN fragment, the cyan base is the PAM region, and the downward arrow (ꜜ) is the theoretical cleavage site.

## Supplementary material 5: The *LacZ* gene in BMLacZ was inserted into the fluorescent gene.

5'…TAGGTCACGTTGGTGTAGATGGGCGCATCGTAACCGTGCATCTGCCAGTTTGAGGGGACGACGACAGTATCGGTGTTGATACAACCATAAAATGATAATTACACCCATAAATTGATAATTATCACACCCATAAATTGATATTGCCTCTTCATGGTCTAAACTTCAGTAAGTTTACGACATTTTCCTCGAGGTCATTTCCGGGGATCCATGGAGAAAAAAATCACTGGATATACCACCGTTGATATATCCCAATGGCATCGTAAAGAACATTTTGAGGCATTTCAGTCAGTTGCTCAATGTACCTATAACCAGACCGTTCAGCTGGATATTACGGCCTTTTTAAAGACCGTAAAGAAAAATAAGCACAAGTTTTATCCGGCCTTTATTCACATTCTTGCCCGCCTGATGAATGCTCATCCGGAGTTCCGTATGGCAATGAAAGACGGTGAGCTGGTGATATGGGATAGTGTTCACCCTTGTTACACCGTTTTCCATGAGCAAACTGAAACGTTTTCATCGCTCTGGAGTGAATACCACGACGATTTCCGGCAGTTTCTACACATATATTCGCAAGATGTGGCGTGTTACGGTGAAAACCTGGCCTATTTCCCTAAAGGGTTTATTGAGAATATGTTTTTCGTCTCAGCCAATCCCTGGGTGAGTTTCACCAGTTTTGATTTAAACGTGGCCAATATGGACAACTTCTTCGCCCCCGTTTTCACTATGGGCAAATATTATACGCAAGGCGACAAGGTGCTGATGCCGCTGGCGATTCAGGTTCATCATGCCGTTTGTGATGGCTTCCATGTCGGCAGAATGCTTAATGAATTACAACAGTACTGCGATGAGTGGCAGGGCGGGGCGTAATTTTTTTAAGGCAGTTATTGGTGCCCTTCTAGAGTCTTGACGGCTAGCTCAGTCCTAGGTACAGTGCTAGCTACTAGAGAAAGAGGAGAAATACTAGATGGTTTCTAAGGGTGAAGAGAACAACATGGCTATTATTAAGGAGTTCATGCGTTTTAAGGTCCACATGGAGGGTTCCGTTAACGGTCATGAATTTGAAATTGAGGGTGAGGGTGAGGGTAGACCATACGAAGCTTTTCAAACTGCTAAGTTGAAGGTCACCAAGGGTGGTCCATTGCCATTTGCTTGGGATATTTTGTCTCCACAATTTATGTACGGTTCCAAGGTTTACATCAAGCACCCAGCTGATATTCCAGATTACTTTAAGTTGTCCTTCCCAGAGGGTTTTAGATGGGAAAGAGTTATGAACTTTGAGGACGGTGGTATTATCCACGTTAACCAAGATTCTTCCTTGCAGGATGGTGTTTTTATTTACAAGGTCAAGTTGCGTGGTACCAACTTTCCATCTGATGGTCCAGTTATGCAAAAGAAGACTATGGGTTGGGAAGCTTCTGAAGAAAGAATGTACCCAGAAGATGGTGCTTTGAAGTCTGAAATTAAGAAGCGTTTGAAGTTGAAGGACGGCGGTCATTACGCTGCTGAAGTTAAGACTACTTACAAGGCTAAGAAGCCAGTTCAATTGCCAGGTGCTTACATTGTTGATATTAAGTTGGACATCGTCTCCCACAACGAGGATTACACTATTGTTGAACAGTACGAGAGAGCCGAAGGTAGACATTCTACTGGTGGTATGGATGAATTGTACAAGTAAGCAGATCTCAATTGGATATCGGCCGGCCACGCGATCGCTGACGTCGGTACCCTCGAGTCTGGTAAAGAAACCGCTGCTGCGAAATTTGAACGCCAGCACATGGACTCGTCTACTAGCGCAGCTTAATTAACCTAGGCTGCTGCCACCGCTGAGCAATAACTAGCATAACCCCTTGGGGCCTCTAAACGGGTCTTGAGGGGTTTTTTGCTGAAAGGAGGAACTATATCCGGATTGGCGAATCTTACTGCAGTAGTTTTGCTGAAATACTCGATTCACAAAAATATCAACTTATGGTTGTTTTGTGAGATATCAATATATGGTTGTTTTGTGGTTAAGTTGCTGATTATAAATAATTATTAAATATCACTTTATGGTTGCATCAACAATCGGCCTCAGGAAGATCGCACTCCAGCCAGCTTTCCGGCACCGCTTCTGG…3'

Note: The green background is *LacZ* gene fragment, the green font is the chloramphenicol resistance gene, the gray background is J23100 constitutive promoter and ribosome binding site fragment, the red font is the *mApple* gene, the yellow base fragment is the gRAN fragment, and the cyan base is the PAM region.

# Supplementary Figures


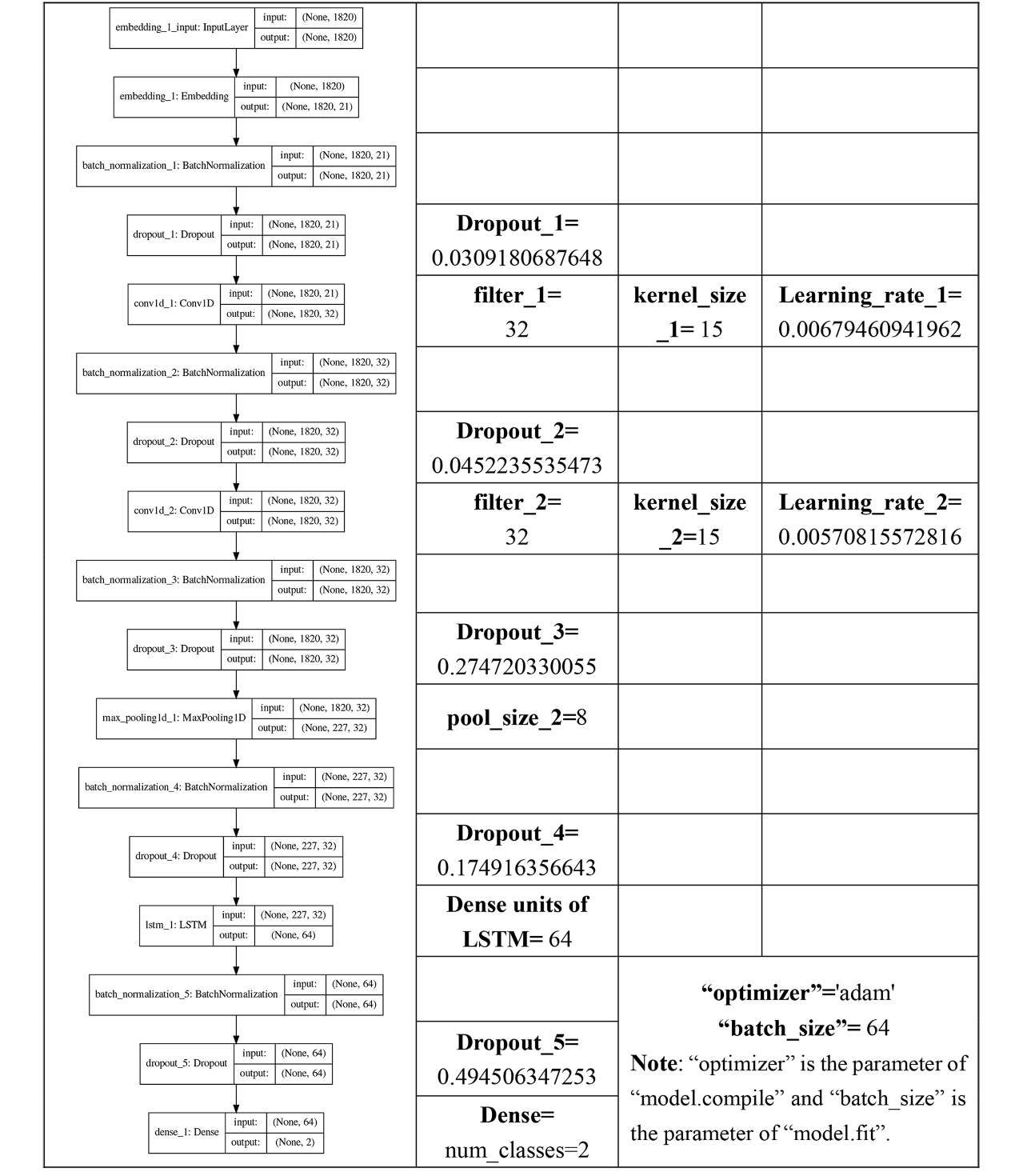


**Figure S1: The construction of CasMiner model.** The left side is the model structure flow chart of CasMiner, and the right side is the parameters adjusted and used in the model training process.


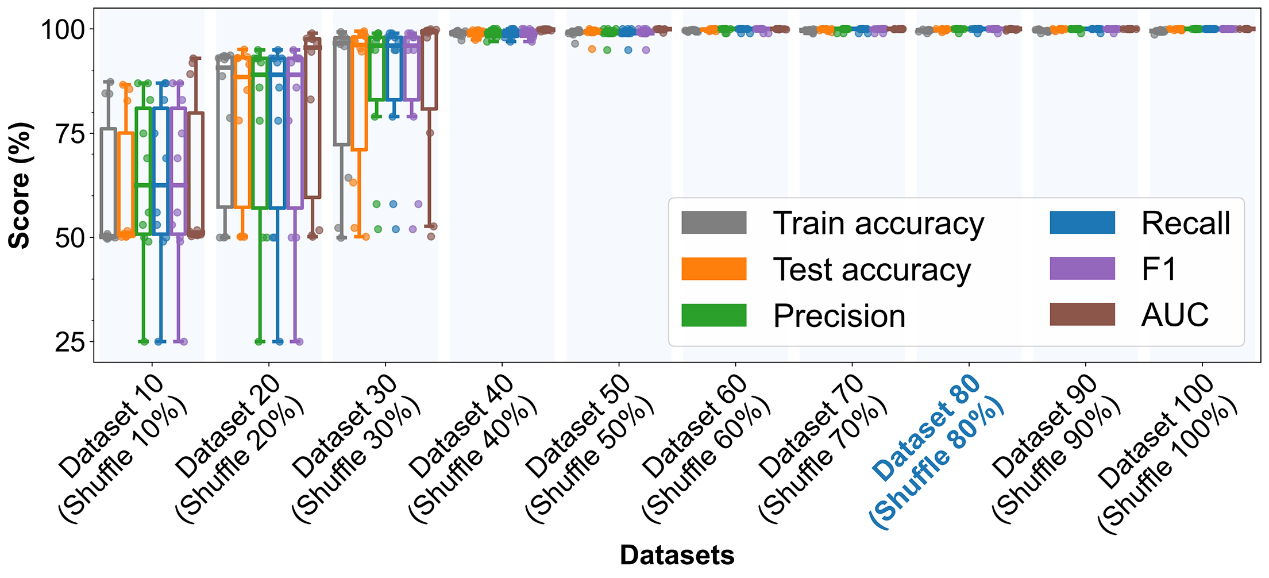


**Figure S2: Model performance parameters.** Statistical analysis of the parameters of the models constructed by different shuffle percentage datasets. The evaluation parameters include Train accuracy, Test accuracy, Precision, Recall, F1 and AUC.


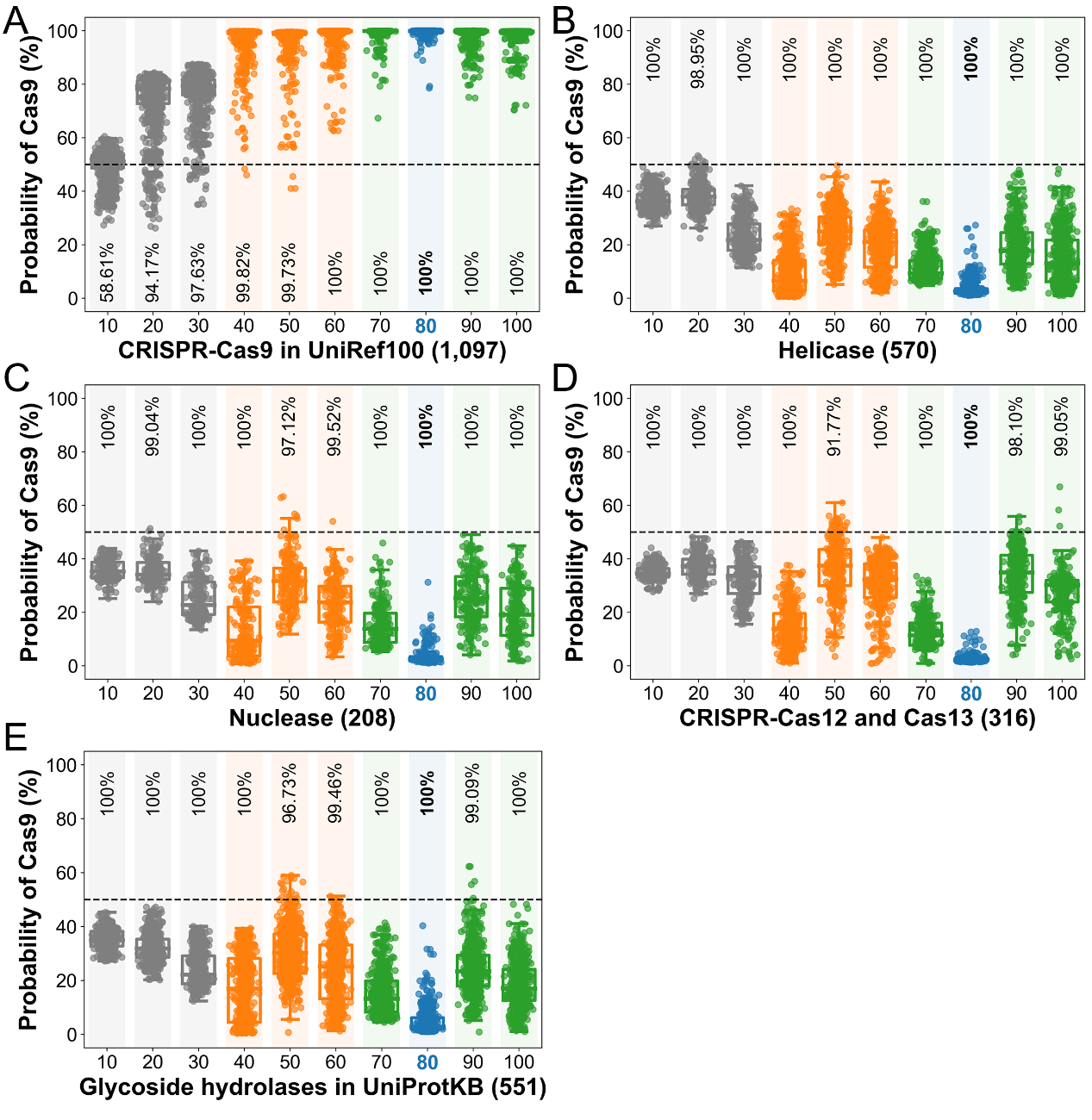


**Figure S3:** **The prediction results of five datasets.** All the models were used to predict and evaluate **A**) the Cas9 dataset of UniRef100 except UniRef90, **B**) the nuclease dataset, **C**) the helicase dataset, **D**) the Cas12-Cas13 dataset, and **E**) the glycoside hydrolase dataset.


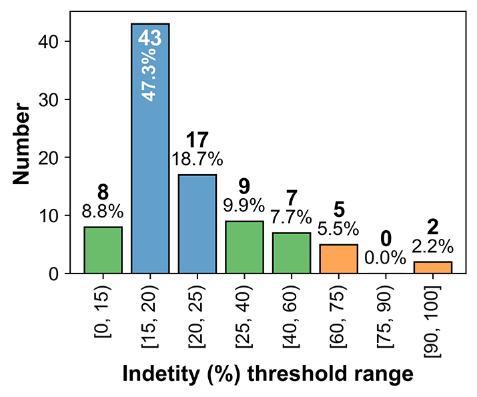


**Figure S4: Identity distribution of pairwise comparison results of sequences.**


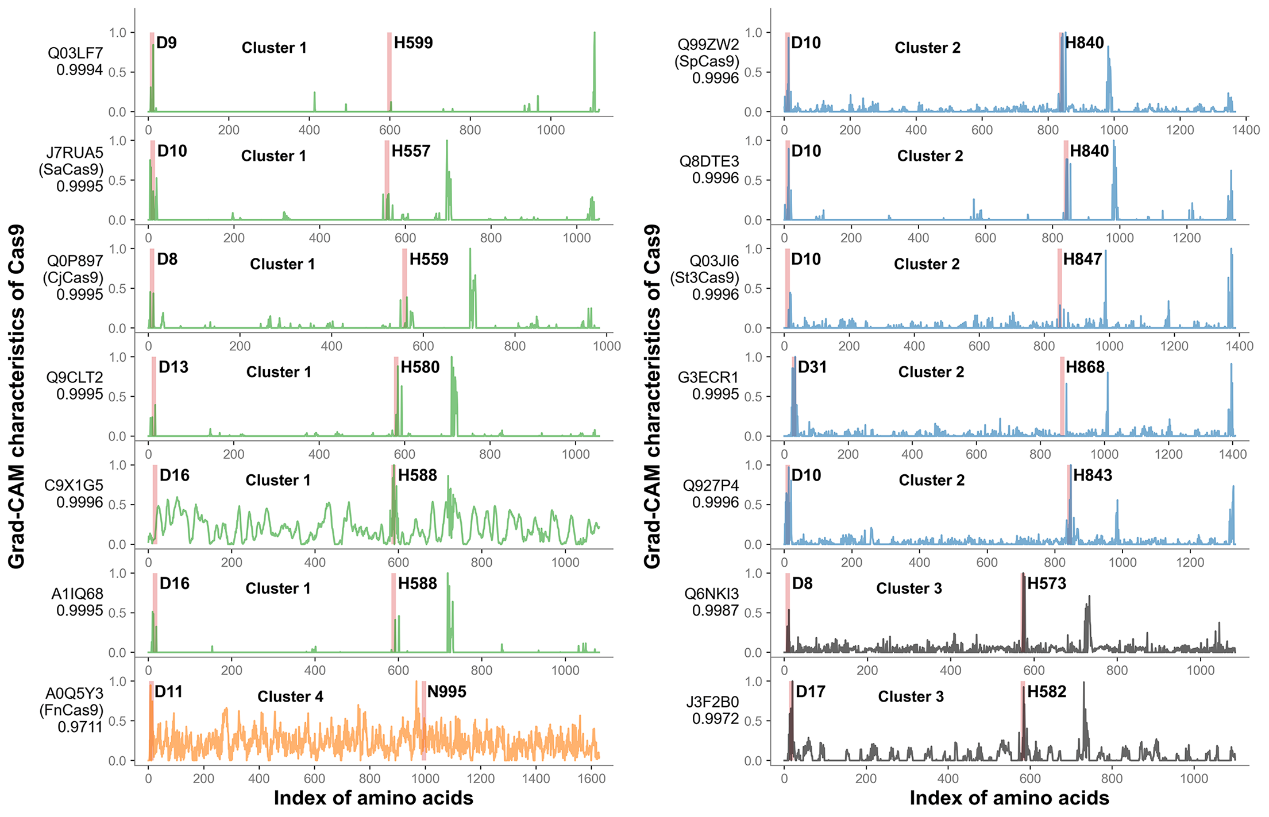


**Figure S5: CasMiner performs prediction and feature extraction on the Reviewed Cas9 sequences.** CasMiner first predicted the Cas9 sequences and extracts the “Cas9 fingerprint” of the Cas9 sequences through Grad-CAM method. In addition, the red bars in the picture represents the active sites annotation of the protein in the UniProt database.


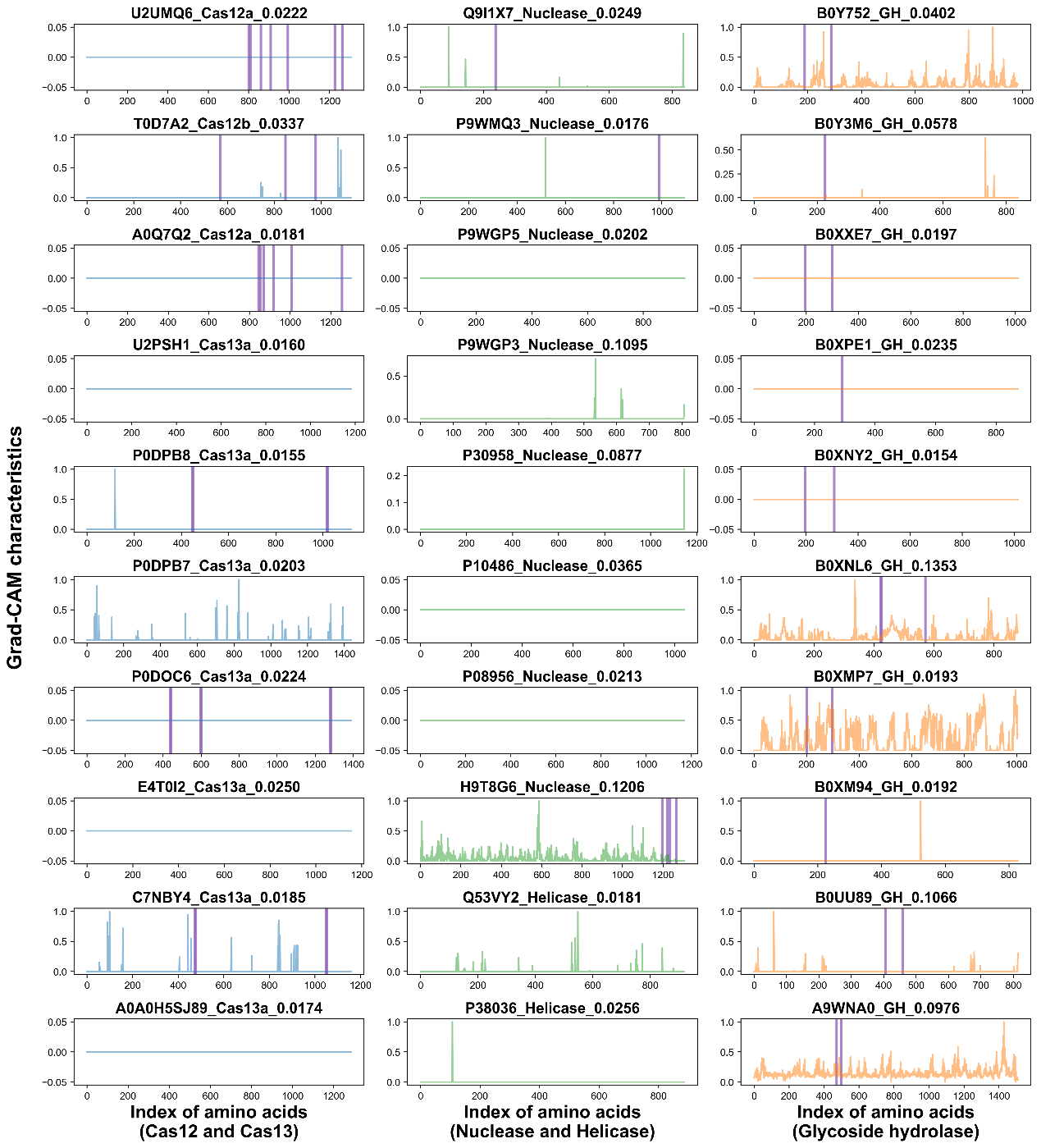


**Figure S6: Grad-CAM feature extraction of target sequences by CasMiner.** CasMiner extracted Grad-CAM characterization of Reviewed Cas12 and Cas13, nuclease, helicase and glycoside hydrolase, respectively. Among them, Cas12, Cas13 and glycoside hydrolase are all reviewed Cas12a, Cas12b and Cas13a. Ten bacterial nucleases and helicases with “Annotation score” equal to 5 and sequence length greater than 801 AA were selected as representative data for feature extraction. In addition, the purple bars in the picture represents the active sites annotation of the protein in the UniProt database.


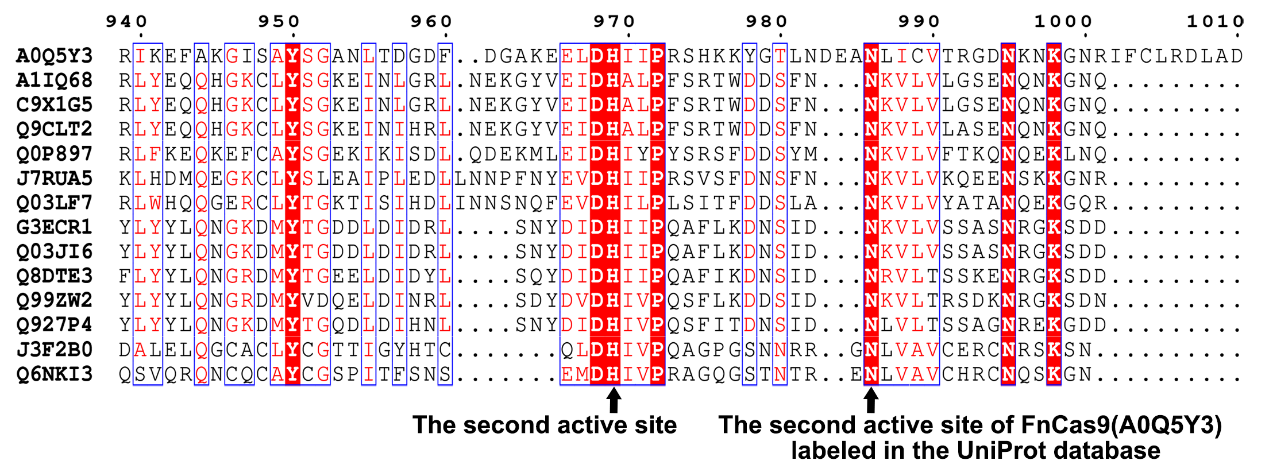


## Figure S7: The multiple sequence alignment of 14 reviewed Cas9 sequences


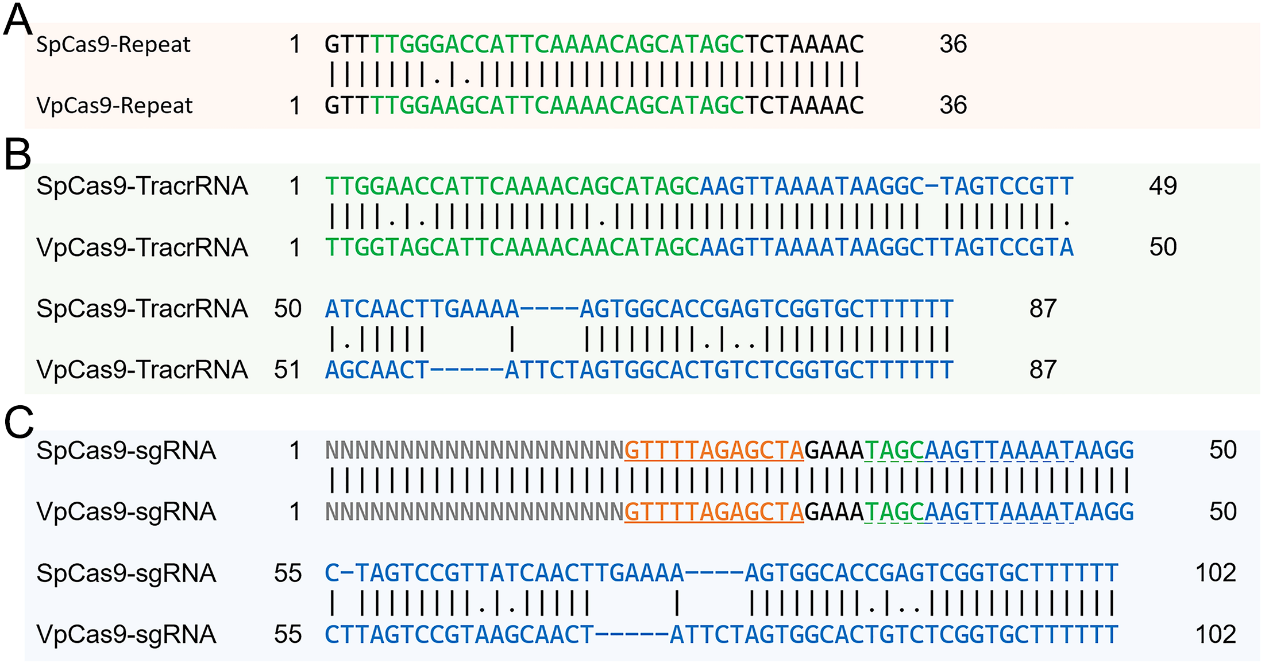


**Figure S8: Global alignment of Repeat, TracrRNA and sgRNA.** All global alignment results were generated using Needle software. **A**) Sequence alignment of SpCas9 and VpCas9 Repeat was performed. **B**) Sequence alignment of SpCas9 and VpCas9 TracrRNA was performed. **C**) Sequence alignment of SpCas9 and VpCas9 sgRNA was performed. In addition, the green base region is the base complementary pairing region of Repeat and TracrRNA, the blue base region is the 3' end sequence of sgRNA, the orange base region is the base complementary pairing fragment that constitutes the first stem loop, and the gray base region is gRNA (Spacer, 20nt).


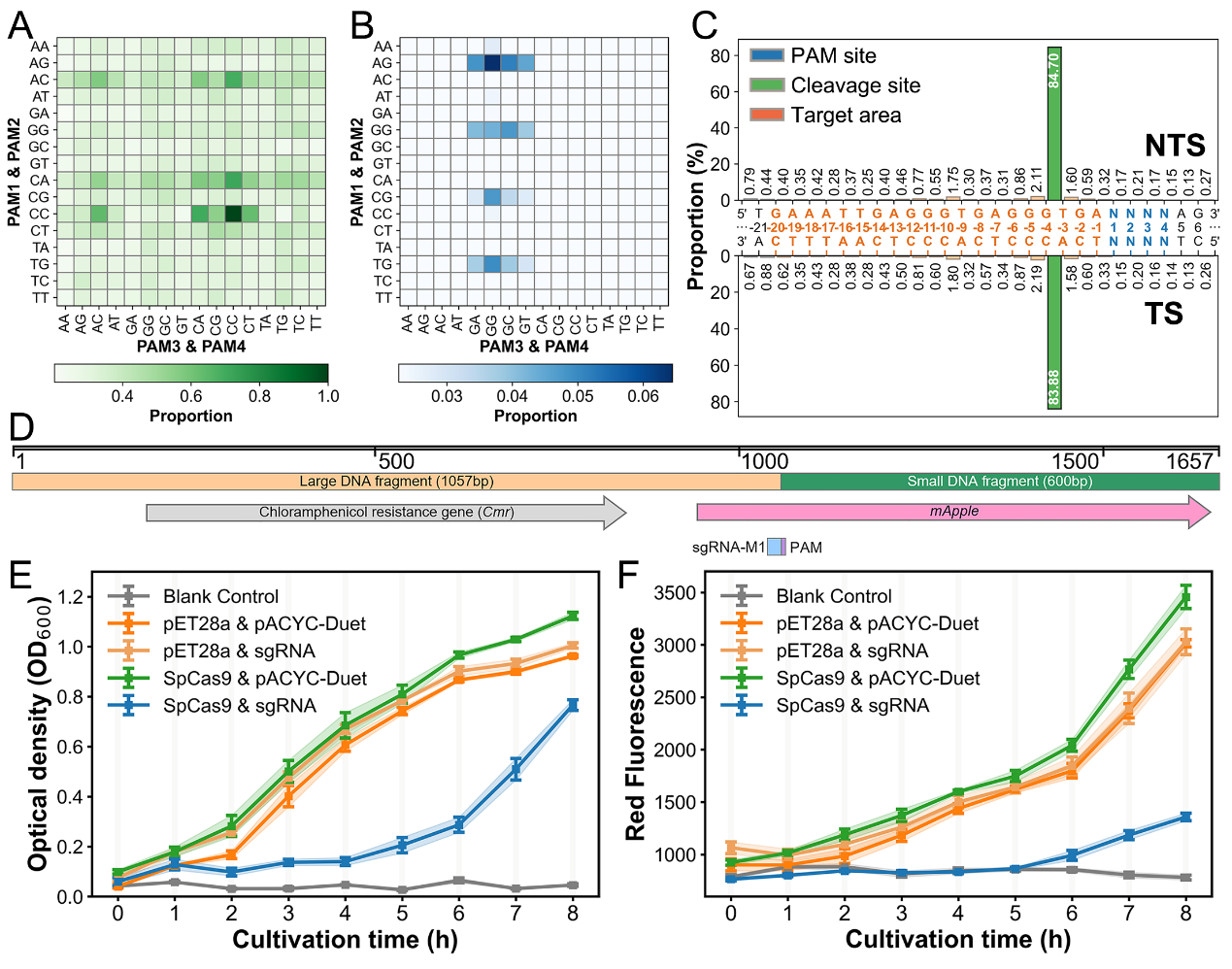


**Figure S9: The PAM library cutting results of spcas 9. A**) concentration distribution matrix of 256 kinds of PAM (NNNN) in PAM library; **B**) The PAM preference matrix of the sequence was obtained by the second-generation sequencing analysis. **C**) Enzymatic digestion analysis based on the sequence data of the second-generation sequencing, where “TS” is “target strand” and “NTS” is “non-target strand”. **D**) Designed to cleave nucleic acid fragments in vitro. **E-F**) Validation of the validity of growth-fluorescence detection platform.


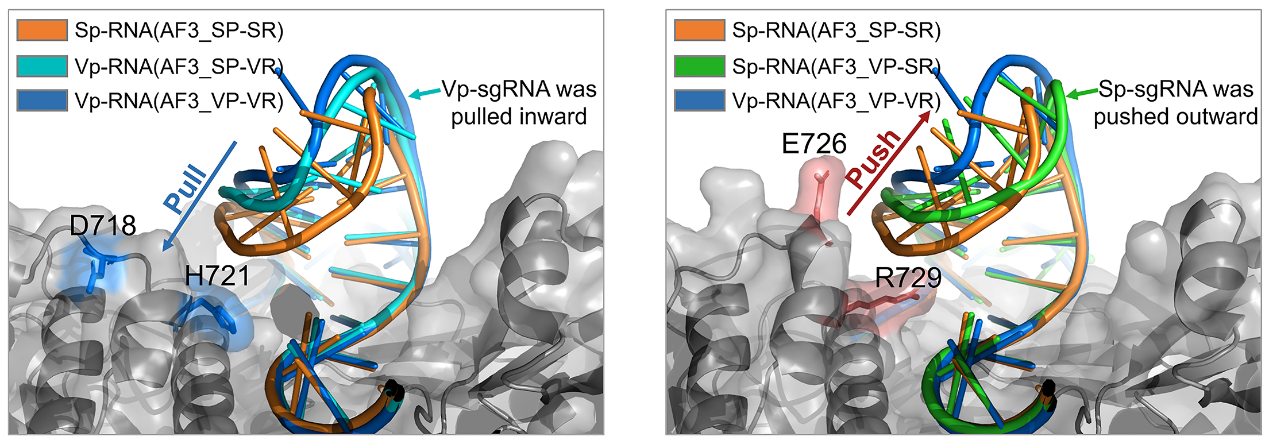


**Figure S10: Structural analysis of sgRNA interactions.** SpCas9 pulls the sgRNA of VpCas9 inward, while VpCas9 pushes the sgRNA of SpCas9 outward.


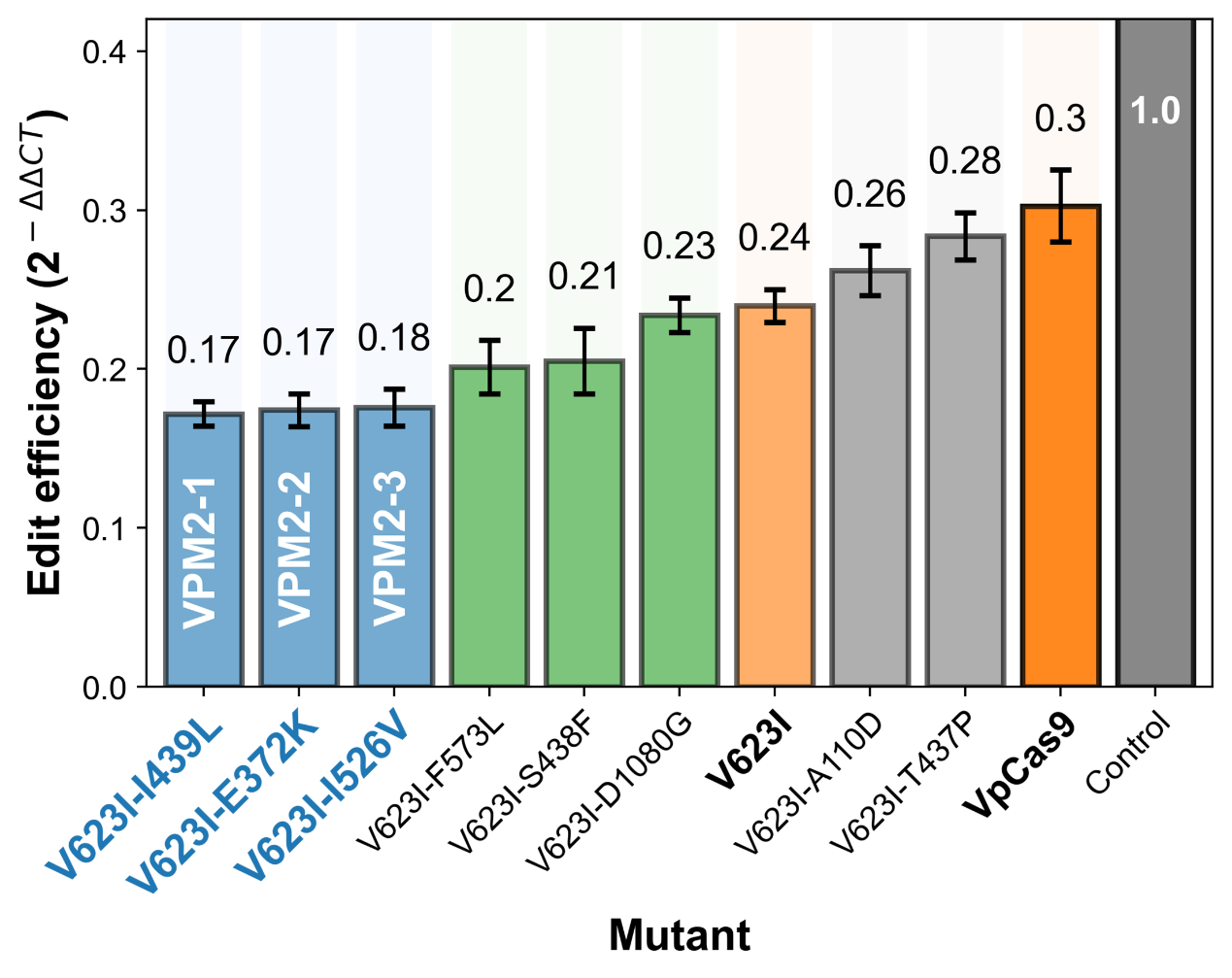


**Figure S11: Activity detection of 8 double point mutations.** Preliminary verification of the cleavage activity of the double point mutant.

**
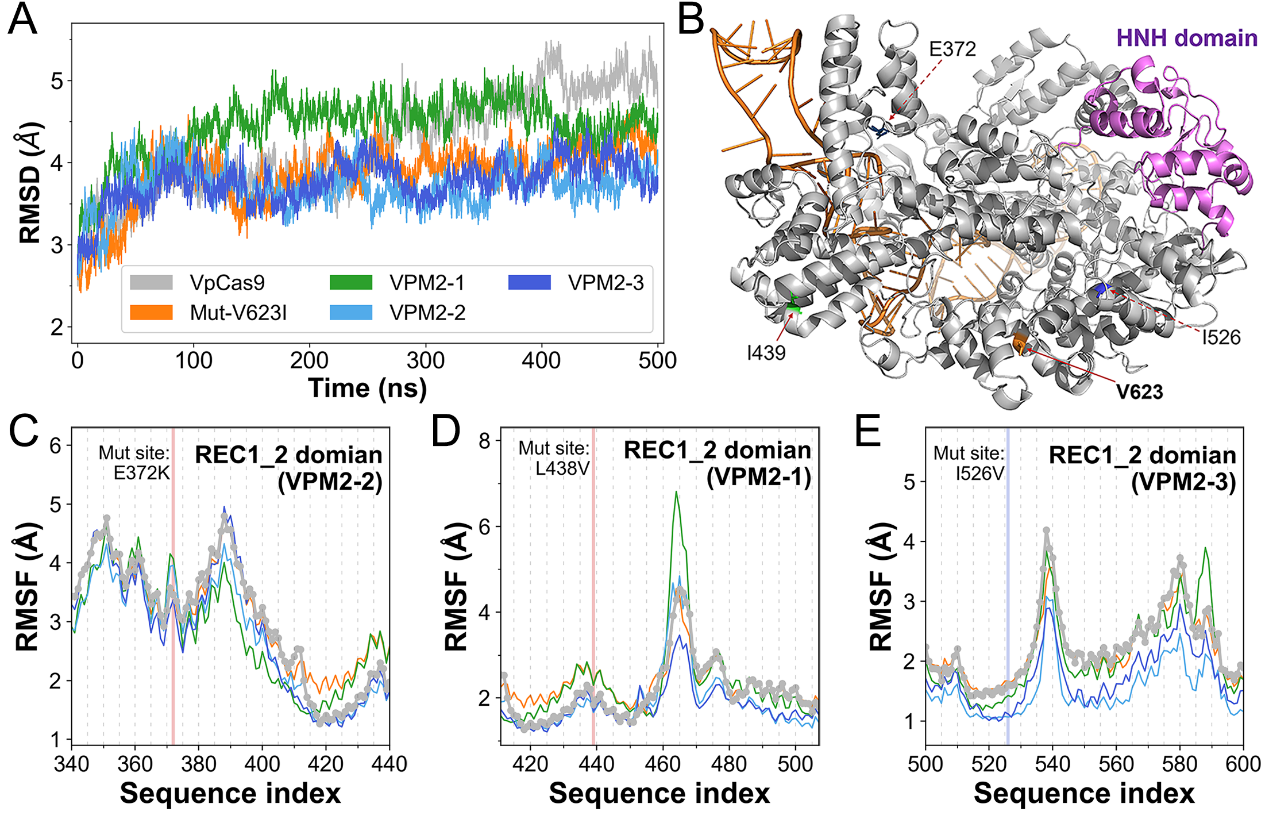
**

**Figure S12: RMSD and RMSF analysis of VpCas9 and its mutants. A**) RMSD analysis of VpCas9 and its mutants; **B**) The distribution of 4 mutation sites in the tertiary structure; **C-E**)The effect of mutation sites of **D**) VPM2-1, **C**) VPM2-2 and **E**) BPM2-3 on RMSF values.


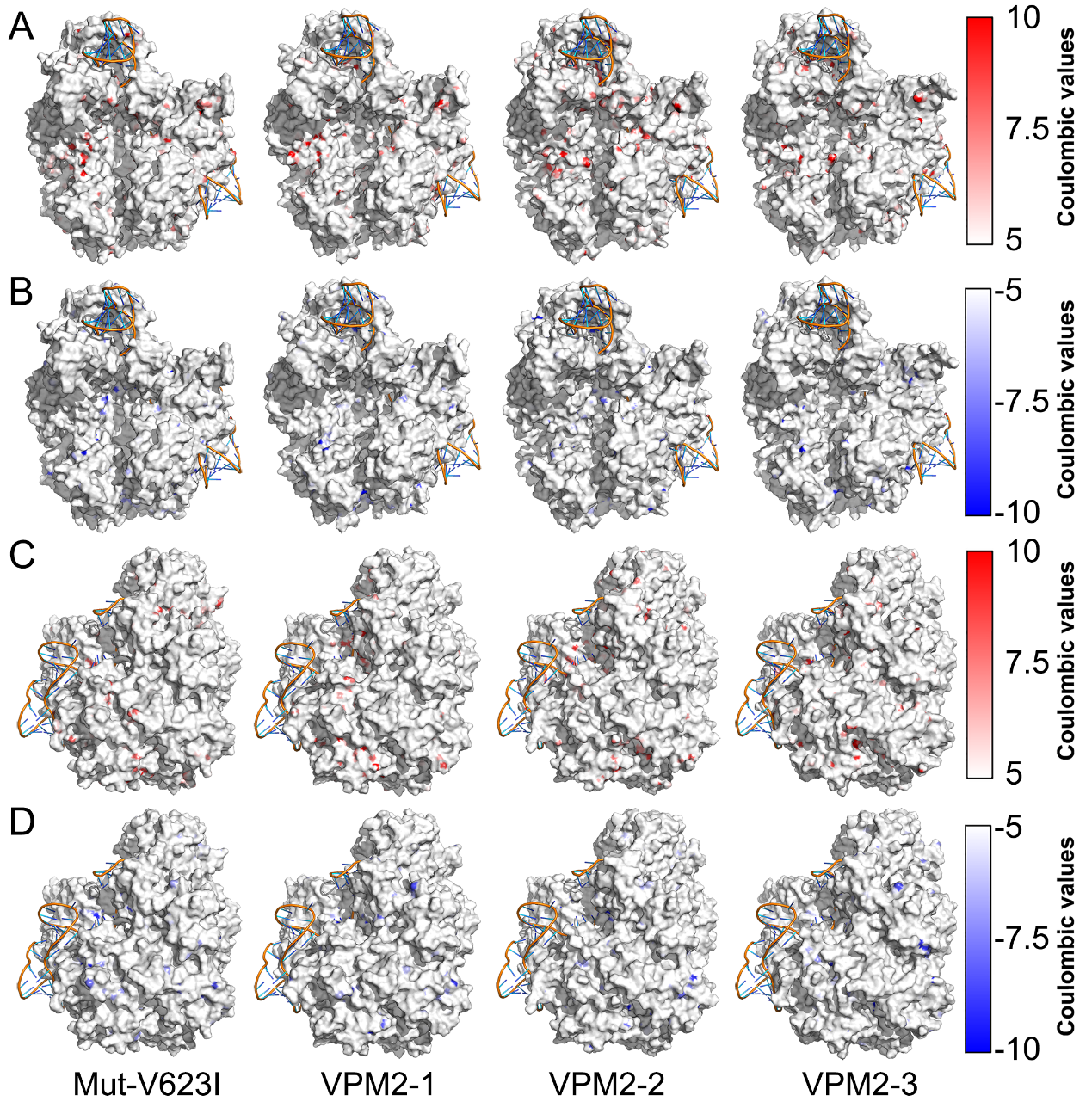


**Figure S13: The electrostatic potential energy changes of the mutants compared with the wild typ. A**) up-regulation and **B**) down-regulation on the front of VpCas9; ESP **C**) up-regulation and **D**) down-regulation on the back of VpCas9.


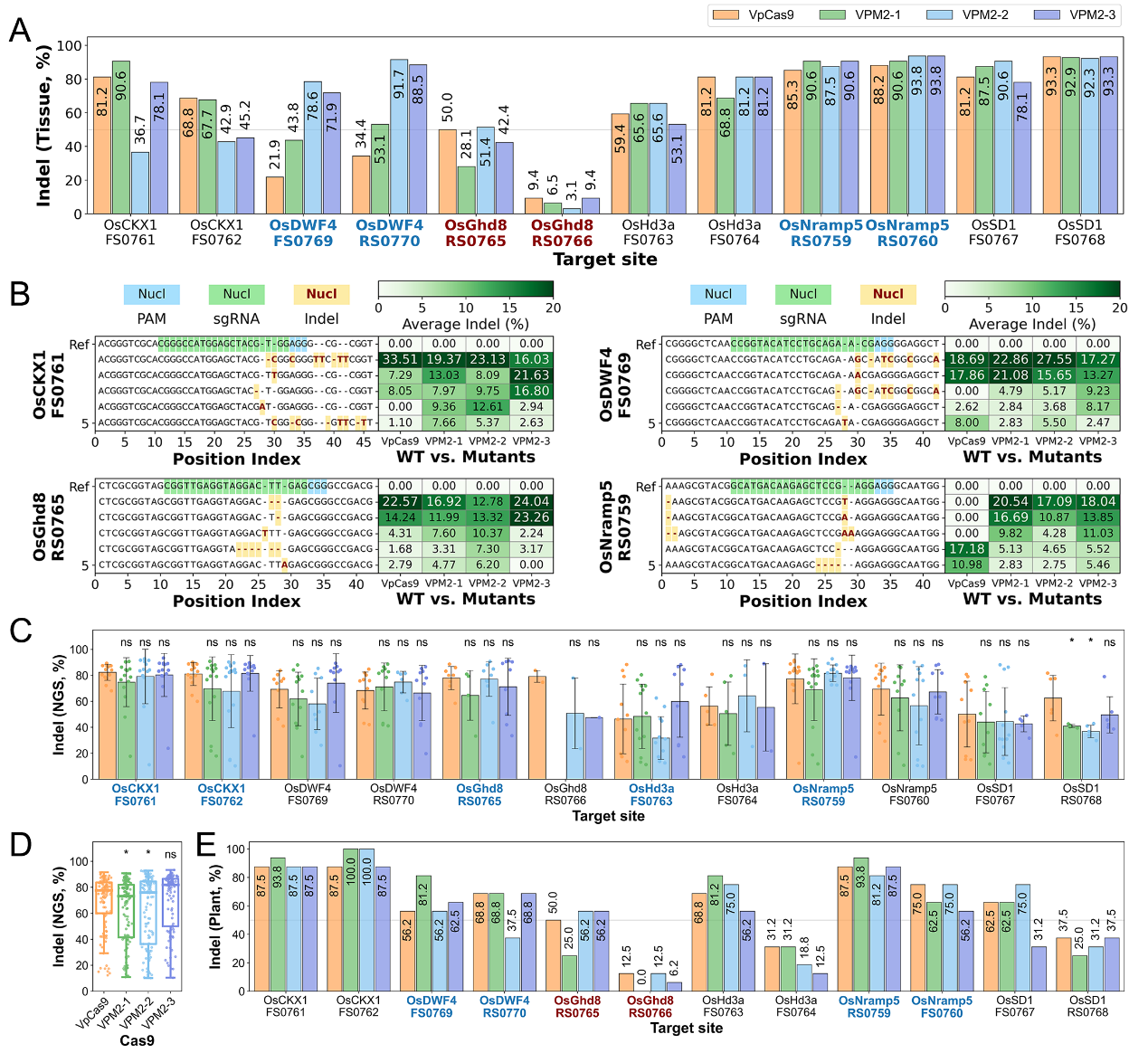


**Figure S14. Genome editing performance of VpCas9 and its mutants in rice callus and T0 plants: efficiency, indel profiles, and edited rates. A**) Edited callus rates for each gene and target site; **B**) Analysis of indel types at four representative target sites. Blue, PAM sequence; green, sgRNA sequence; yellow, modified bases in nuclear genome; **C**) Gene editing efficiency at 12 target sites in T0 plants; **D**) Average gene editing efficiency across all targets in T0 plants; **E**) Edited T0 plant rates for each gene and target site. In panel **B** and **D**, blue-labeled genes denote lower editing rates for VpCas9, while red-labeled genes represent genes with poor editing efficiency (i.e., hard-to-edit targets) for the enzyme. In addition, significance was determined by Two-tailed T-test at the level of ***, p < 0.001, extremely significant. **, p < 0.01, highly significant. *, p < 0.05, significant. ns, not significant.


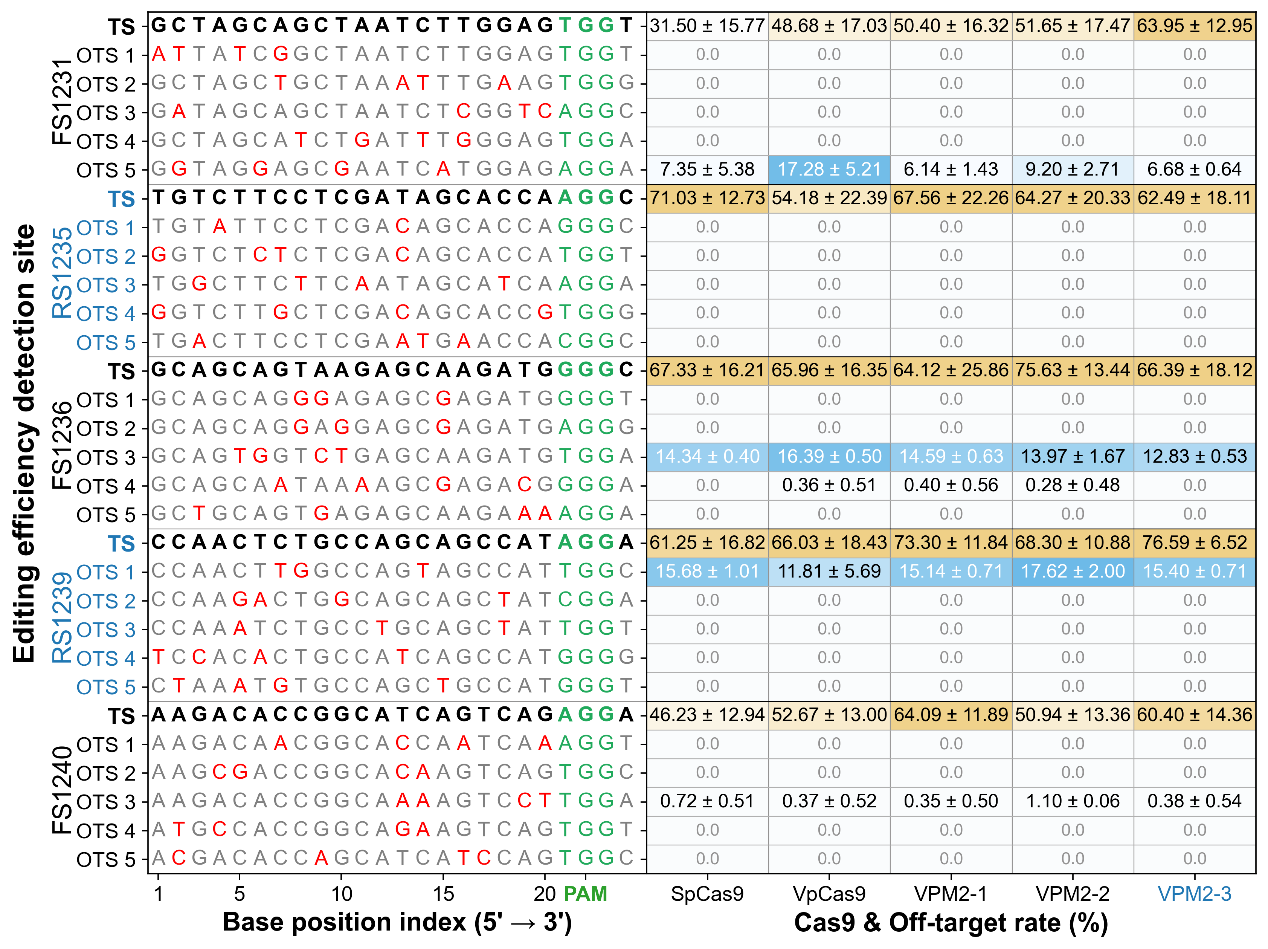


**Figure S15: Editing efficiency of target sites in *Nramp5* and their corresponding off-target sites**


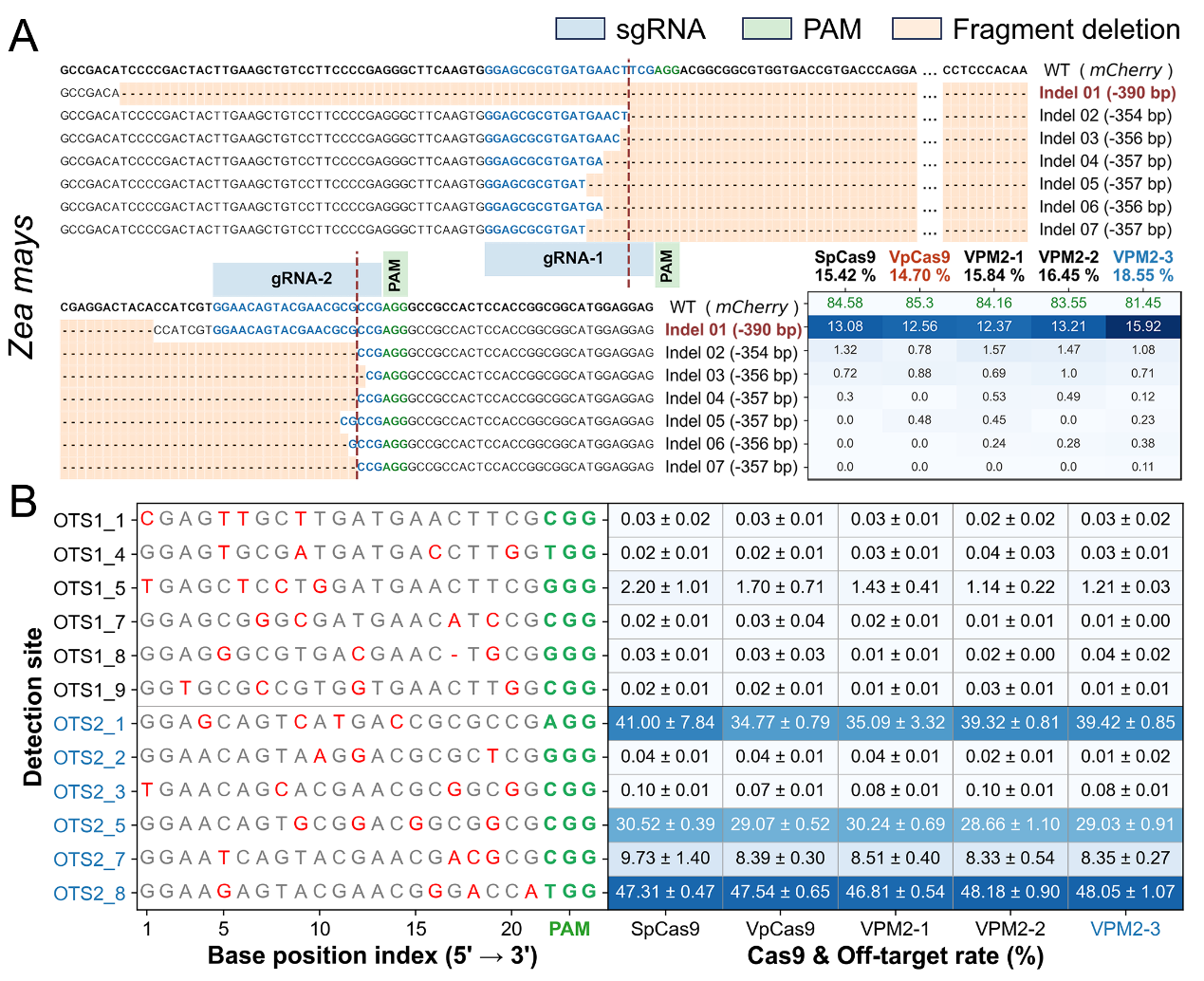


**Figure S16: Fragment knockout results in maize protoplasts and off-target analysis of the corresponding target sites**


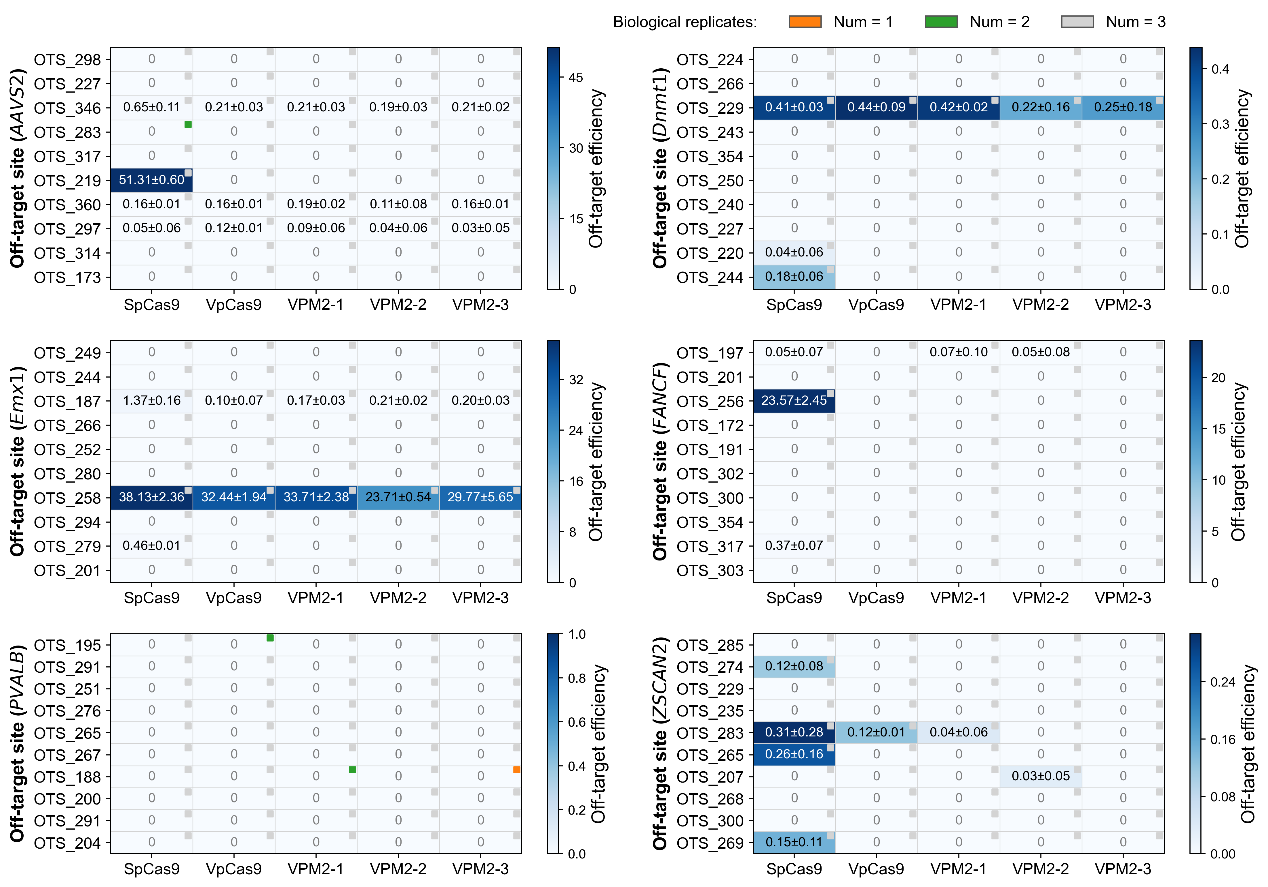


**Figure S17: Off-target analysis of HEK293T.**


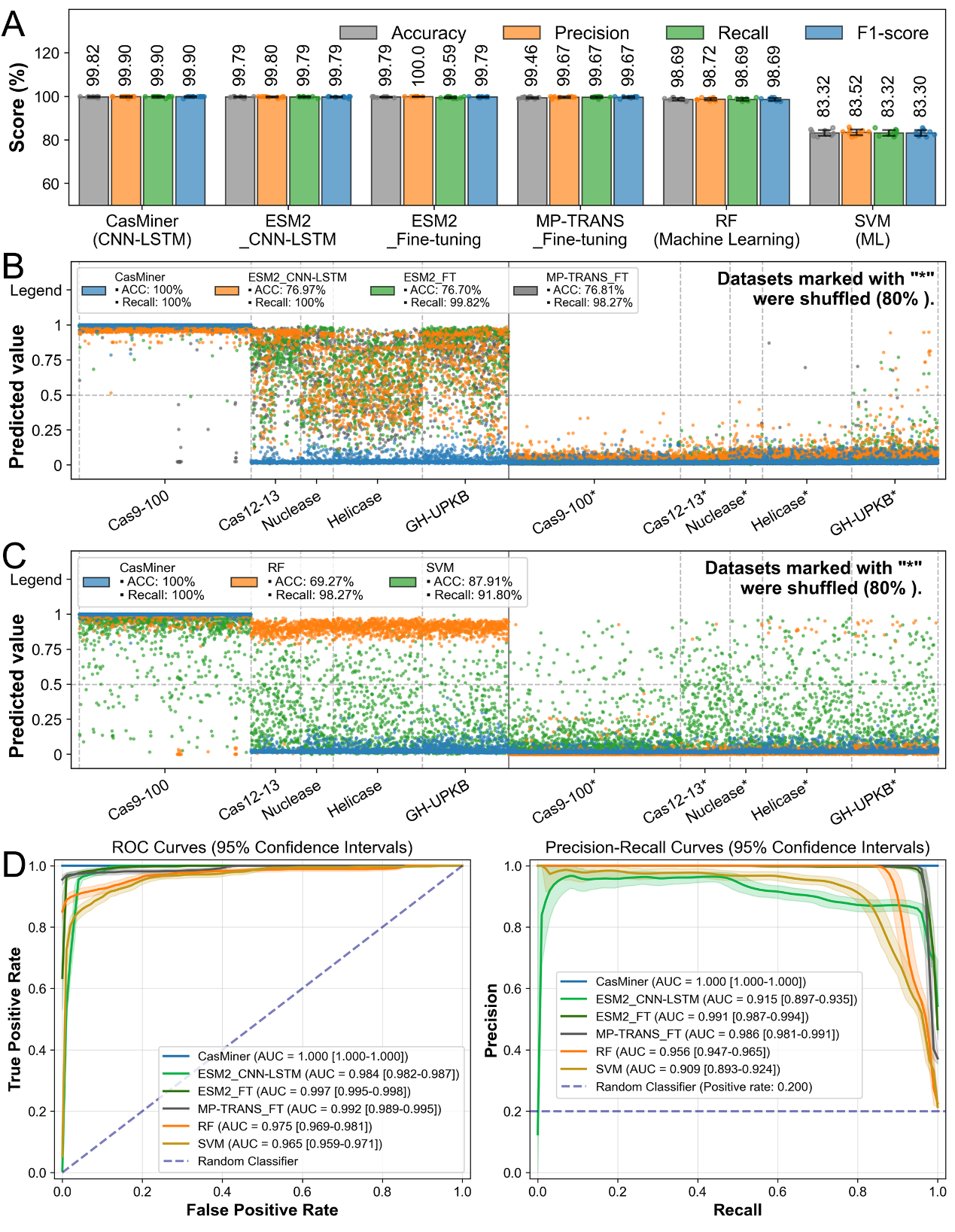


**Figure S18: Evaluation of the generalization ability of CasMiner and other models built on the same training dataset. A)** Performance comparison of model parameters (Accuracy, Precision, Recall, F1-score) among different models; **B)** Comparison of generalization ability between CasMiner and the ESM2-8M encoding followed by CNN-LSTM model (ESM2_CNN-LSTM), the fine-tuned ESM2-8M model (ESM2_FT), and the fine-tuned MP-TRANS model (MP-TRANS_FT); **C)** Comparison of generalization ability between CasMiner and Random Forest (RF) and Support Vector Machine (SVM); **D)** Analysis and evaluation of AUROC and AUPRC for each model under a new dataset.


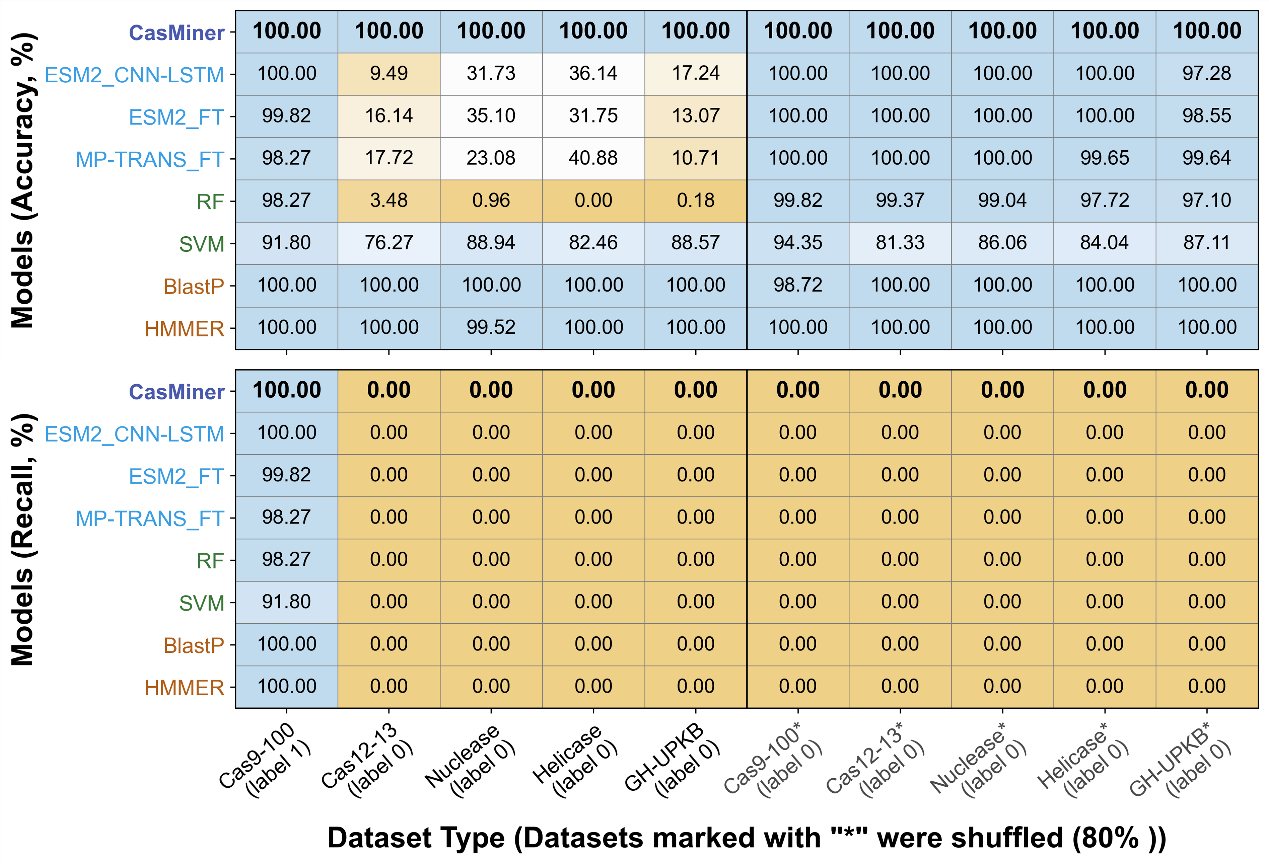


**Figure S19:** Accuracy and Recall evaluation of different methods on 10 independent test datasets.


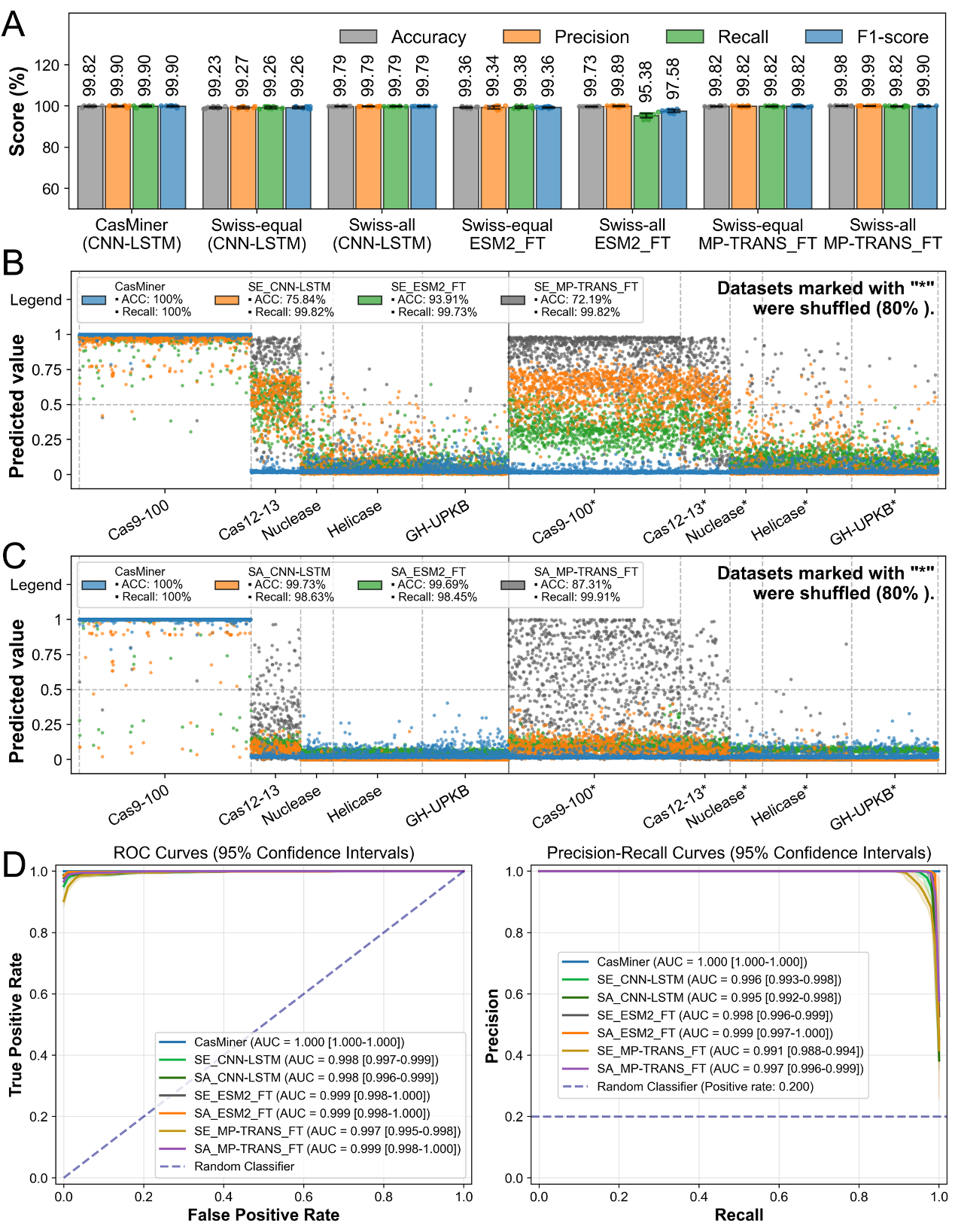


**Figure S20: Evaluation of the generalization ability of CasMiner and other models constructed using SwissProt as the negative dataset. A)** Comparison of model parameters and performance metrics across different models; **B)** Generalization ability comparison under a size-matched SwissProt negative dataset between CasMiner and models including a Keras model with the same framework, a fine-tuned ESM-8M model, and a fine-tuned MP-Trans model; **C)** Generalization ability comparison using the full SwissProt negative dataset between CasMiner and the same set of benchmark models; **D)** Analysis and evaluation of AUROC and AUPRC for each model on a new independent dataset.


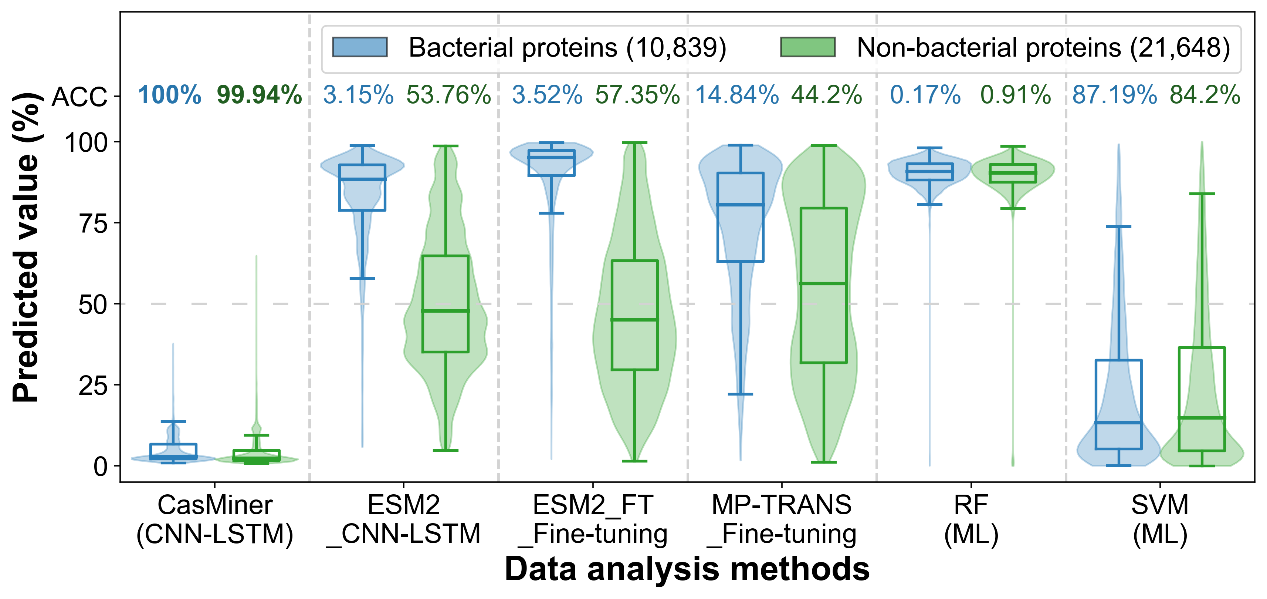


**Figure S21: Large-scale evaluations of CasMiner and other artificial-sequence-based models on the Swiss-all negative (non-Cas9) dataset**


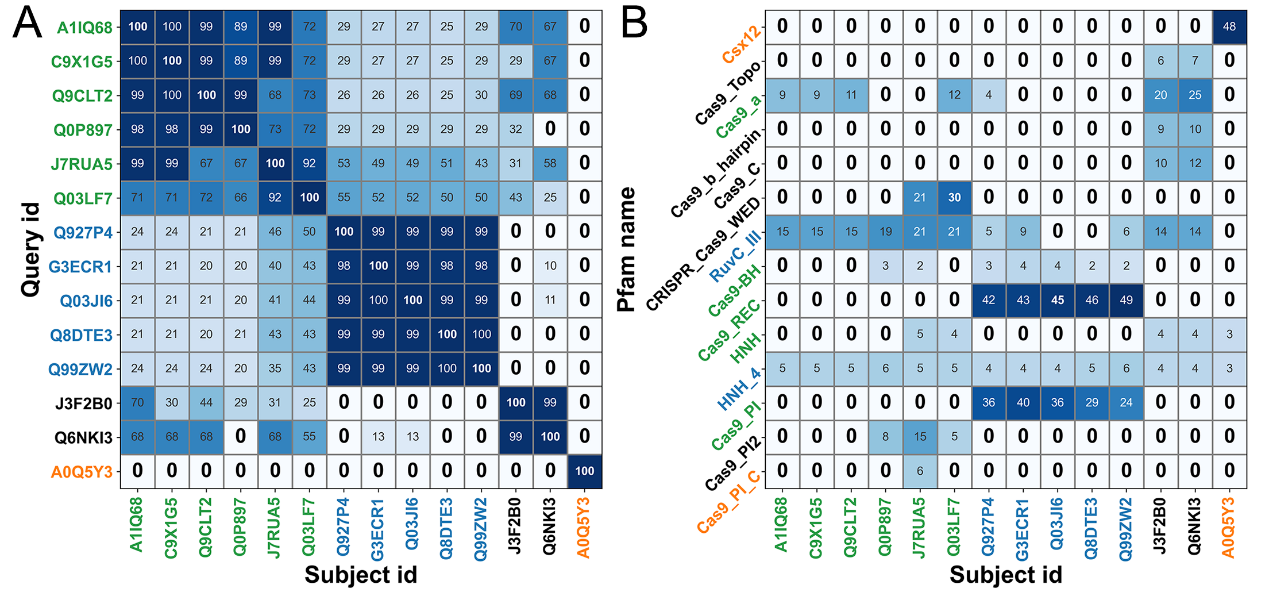


**Figure S22: Evaluation of BlastP and HMMER screens for known Cas9s. A**) Coverage statistics of sequences retrieved by BlastP. Score of 0 indicates not retrieved; **B**) Retrieved sequence length versus total length in hmmscan screen with 14 pFam domains. Score of 0 indicates no sequence was retrieved.


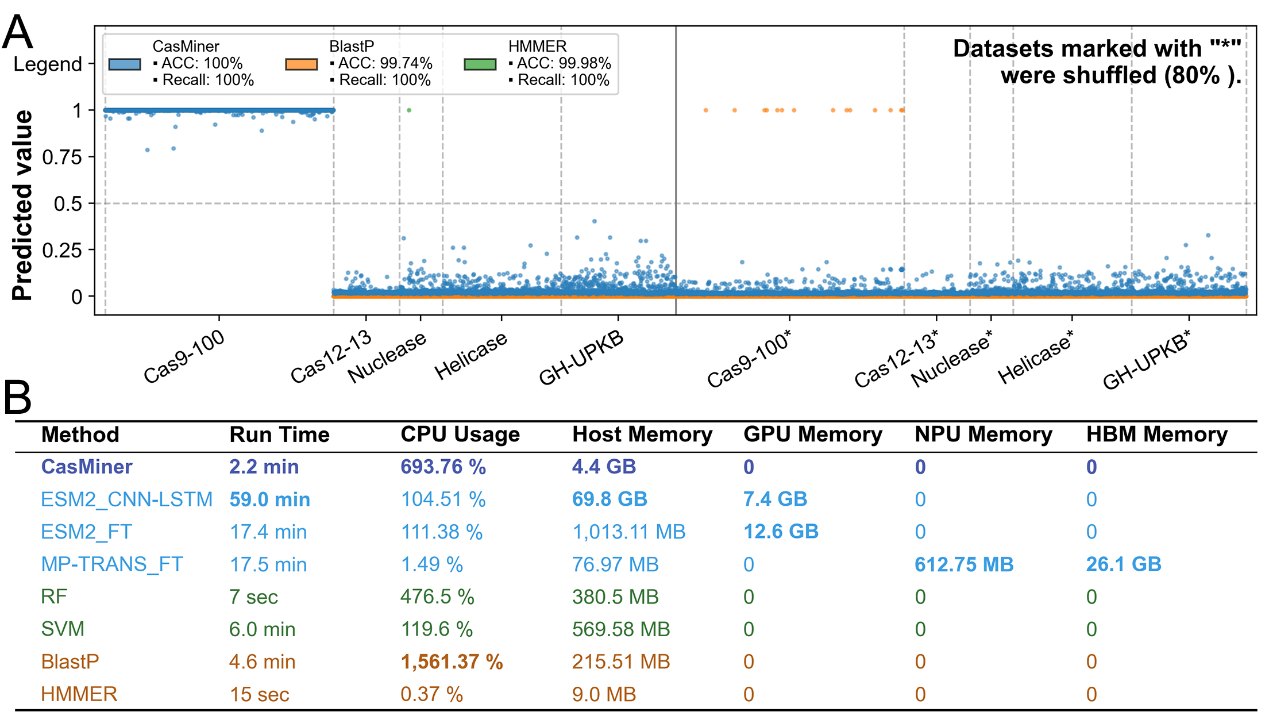


**Figure S23. Performance and resource consumption benchmarks for CasMiner. A)** Retrieval performance comparison of CasMiner, BLASTP, and HMMER on ten datasets. **B)** Comparison of computational resources (e.g., time and memory) used by CasMiner, conventional tools, and other AI models.

# Supplementary Tables

## Table S1: Pfam included in 14 reviewed Cas9 sequences

See the **sheet 1** table in “CasMiner_ST.xlsx” for more information.

## Table S2: Repeat analysis of potential Cas9 based on CasMiner prediction

See the **sheet 2** table in “CasMiner_ST.xlsx” for more information.

## Table S3: Design of gRNA targeting *mApple* gene by sgRNAcas9 software

See the **sheet 3** table in “CasMiner_ST.xlsx” for more information.

## Table S4: Grad-CAM Matrix of VpCas9.

See the **sheet 4** table in “CasMiner_ST.xlsx” for more information.

## Table S5: The PSAP (position-specific amino acid probabilities) matrix of VpCas9

See the **sheet 5** table in “CasMiner_ST.xlsx” for more information.

## Table S6: Conjoint analysis of GradCAM and PSAP matrix of VpCas9 and mutant design.

See the **sheet 6** table in “CasMiner_ST.xlsx” for more information.

## Table S7: The area in each contour of FEL

See the **sheet 7** table in “CasMiner_ST.xlsx” for more information.

## Table S8: Detection of genome editing efficiency in rice calluses

See the **sheet 8** table in “CasMiner_ST.xlsx” for more information.

## Table S9: Detection of off-target efficiency of genome editing in rice calluses

See the **sheet 9** table in “CasMiner_ST.xlsx” for more information.

## Table S10: Proportion of rice calluses with positive gene editing

See the **sheet 10** table in “CasMiner_ST.xlsx” for more information.

## Table S11: Detection of genome editing efficiency in rice T0 plants

See the **sheet 11** table in “CasMiner_ST.xlsx” for more information.

## Table S12: Proportion of rice T0 plants with positive gene editing

See the **sheet 12** table in “CasMiner_ST.xlsx” for more information.

## Table S13: Detection of genome editing efficiency at the *Nramp5* locus in rice calluses

See the **sheet 13** table in “CasMiner_ST.xlsx” for more information.

## Table S14: Detection of genome editing off-target efficiency associated with the *Nramp5* locus in rice calluses

See the **sheet 14** table in “CasMiner_ST.xlsx” for more information.

## Table S15: Detection of genome editing efficiency in maize protoplasts

See the **sheet 15** table in “CasMiner_ST.xlsx” for more information.

## Table S16: Detection of genome off-target editing efficiency in maize protoplasts

See the **sheet 16** table in “CasMiner_ST.xlsx” for more information.

## Table S17: Detection of genome editing efficiency in HEK293T cell

See the **sheet 17** table in “CasMiner_ST.xlsx” for more information.

## Table S18: Detection of genome off-target editing efficiency in HEK293T cell

See the **sheet 18** table in “CasMiner_ST.xlsx” for more information.

## Table S19: Evaluation of model generalization ability on the synthetic dataset

See the **sheet 19** table in “CasMiner_ST.xlsx” for more information.

## Table S20: Valuation of model generalization ability on the real dataset based on SwissProt

See the **sheet 20** table in “CasMiner_ST.xlsx” for more information.

## Table S21: Large-scale evaluations of CasMiner and other artificial-sequence-based models on the Swiss-all negative (non-Cas9) dataset

See the **sheet 21** table in “CasMiner_ST.xlsx” for more information.

## Table S22: Benchmark comparison of computational resource consumption across different methods

See the **sheet 22** table in “CasMiner_ST.xlsx” for more information.

## Table S23: Primers for mutant construction

See the **sheet 23** table in “CasMiner_ST.xlsx” for more information.

## Table S24: Primers for qPCR detection

See the **sheet 24** table in “CasMiner_ST.xlsx” for more information.

## Table S25: Primers for construction of rice genome editing plasmid

See the **sheet 25** table in “CasMiner_ST.xlsx” for more information.

## Table S26: Primers for detection of rice genome editing efficiency

See the **sheet 26** table in “CasMiner_ST.xlsx” for more information.

## Table S27: Rice genome editing off-target sites and primers for efficiency detection

See the **sheet 27** table in “CasMiner_ST.xlsx” for more information.

## Table S28: Primers for genome editing efficiency at the *Nramp5* locus in rice calluses

See the **sheet 28** table in “CasMiner_ST.xlsx” for more information.

## Table S29: primers for genome editing off-target efficiency associated with the *Nramp5* locus in rice calluses

See the **sheet 29** table in “CasMiner_ST.xlsx” for more information.

## Table S30: On- and off-target information as well as corresponding primer sequences in maize

See the **sheet 30** table in “CasMiner_ST.xlsx” for more information.

## Table S31: On-target and off-target information as well as corresponding primer sequences in HEK293T cell

See the **sheet 31** table in “CasMiner_ST.xlsx” for more information.
